# Western High-Fat Diet Consumption during Adolescence Increases Susceptibility to Traumatic Stress while Selectively Disrupting Hippocampal and Ventricular Volumes

**DOI:** 10.1523/ENEURO.0125-16.2016

**Published:** 2016-11-08

**Authors:** Priya Kalyan-Masih, Julio David Vega-Torres, Christina Miles, Elizabeth Haddad, Sabrina Rainsbury, Mohsen Baghchechi, Andre Obenaus, Johnny D. Figueroa

**Affiliations:** 1 Center for Health Disparities and Molecular Medicine, Loma Linda University School of Medicine, Loma Linda, California 92350; 2 Physiology Division, Department of Basic Sciences, Loma Linda University School of Medicine, Loma Linda, California 92350; 3 Department of Pediatrics, Loma Linda University School of Medicine, Loma Linda, California 92350

**Keywords:** behavior, high-fat diet, hippocampus, imaging, obesity, PTSD

## Abstract

Psychological trauma and obesity co-occur frequently and have been identified as major risk factors for psychiatric disorders. Surprisingly, preclinical studies examining how obesity disrupts the ability of the brain to cope with psychological trauma are lacking. The objective of this study was to determine whether an obesogenic Western-like high-fat diet (WD) predisposes rats to post-traumatic stress responsivity. Adolescent Lewis rats (postnatal day 28) were fed *ad libitum* for 8 weeks with either the experimental WD diet (41.4% kcal from fat) or the control diet (16.5% kcal from fat). We modeled psychological trauma by exposing young adult rats to a cat odor threat. The elevated plus maze and the open field test revealed increased psychological trauma-induced anxiety-like behaviors in the rats that consumed the WD when compared with control animals 1 week after undergoing traumatic stress (*p* < 0.05). Magnetic resonance imaging showed significant hippocampal atrophy (20% reduction) and lateral ventricular enlargement (50% increase) in the animals fed the WD when compared with controls. These volumetric abnormalities were associated with behavioral indices of anxiety, increased leptin and FK506-binding protein 51 (FKBP51) levels, and reduced hippocampal blood vessel density. We found asymmetric structural vulnerabilities to the WD, particularly the ventral and left hippocampus and lateral ventricle. This study highlights how WD consumption during adolescence impacts key substrates implicated in post-traumatic stress disorder. Understanding how consumption of a WD affects the developmental trajectories of the stress neurocircuitry is critical, as stress susceptibility imposes a marked vulnerability to neuropsychiatric disorders.

## Significance Statement

Psychological trauma and obesity are highly prevalent in adolescents. However, preclinical studies investigating how obesity alters psychological trauma responsivity are lacking. In this study, we report that adolescent rats consuming a typical obesogenic Western high-fat diet (WD) display exaggerated post-traumatic stress reactions during early adulthood, along with increased hippocampal expression of the post-traumatic stress biomarker FKBP51. We reveal distinctive hippocampal and ventricular volumetric abnormalities potentially underlying the effects of the WD in maladaptive stress responsiveness. We anticipate that this study will inform the path to needed biomarkers and interventions for improving the quality of stress and anxiety management, particularly in a growing overweight and obese population.

## Introduction

Childhood and adolescent psychological trauma is referred as “the hidden epidemic” (Lanius et al., 2010). Children and adolescents are exposed to a wide range of traumatic events, including abuse, domestic violence, traumatic loss and grief, community and school violence, medical trauma, war-zone and refugee trauma, natural disasters, and terrorism (Friedman et al., 2014). Unfortunately, although many youths who experience psychological trauma recover, approximately one-third develop enduring symptoms of anxiety and post-traumatic stress disorder (PTSD; Pine and Cohen, 2002; Merikangas et al., 2010). Adolescents and young adults with PTSD experience emotional distress and may re-experience the trauma through intrusive recollections of the event while avoiding reminders of the trauma. Interestingly, several lines of epidemiological evidence show that PTSD is often accompanied by comorbid overweight and obesity (Scott et al., 2008; Perkonigg et al., 2009; Pagoto et al., 2012; Johannessen and Berntsen, 2013; Maguen et al., 2013; Mitchell et al., 2013; Kubzansky et al., 2014; Bartoli et al., 2015; Smith et al., 2015). While these studies demonstrate that PTSD leads to an increased risk for obesity, limited focus has been placed on understanding how overweight and obesity increase the susceptibility to the effects of stress on the brain and behavior. As the burden of obesity and traumatic stress continues growing at alarming rates, studies are needed to clarify this interplay. Consumption of Western high-fat diets (WDs), which typically are rich in saturated fats, is a major factor contributing to the global obesity epidemic in children and adolescents (Moreno et al., 2010; Ogden et al., 2010). It is now becoming increasingly recognized that dietary consumption of a WD impairs optimal brain development and function, and is associated with adverse cognitive and emotional outcomes later in life (Nilsson et al., 2009; Sellbom and Gunstad, 2012; Francis and Stevenson, 2013). Surprisingly, studies that examine how Western dietary patterns impact the maturation and function of the stress-response neurocircuitry are lacking. The hippocampus (HPC), a brain region traditionally studied for its role in memory and learning, plays important roles in terminating the physiological stress responses via feedback inhibition of the hypothalamic–pituitary–adrenal (HPA) axis (Jacobson and Sapolsky, 1991; Sapolsky, 2001). The sensitivity of this feedback mechanism is regulated via complex interactions among environmental stressors, genes, and epigenetic modifications of glucocorticoid-responsive genomic sites. An important modulator of these interactions is *FKBP5* (Hoeijmakers et al., 2014; Schmidt et al., 2015), which codes for the FK506-binding protein 51 (FKBP51) and has emerged as a promising drug target for its involvement in numerous neuropsychiatric disorders, including PTSD (Binder et al., 2004, 2008; Binder, 2009; Binder et al., 2010; Lavebratt et al., 2010; Ellsworth et al., 2013; Klengel et al., 2013; Alemany et al., 2016; Stamm et al., 2016; Yehuda et al., 2016). Notably, new studies show a strong relationship between the *FKBP5* gene and hippocampal volumes and connectivity (Zobel et al., 2010; Fani et al., 2013; Córdova-Palomera et al., 2016). Thus, FKBP51 may play a role in predisposing the brain to traumatic stress by virtue of regulating the structural integrity of the hippocampus.

Here, we hypothesized that the consumption of a WD during adolescence confers vulnerability to PTSD-related behaviors following traumatic stress, while altering the hippocampal molecular and structural landscape. One important function of the hippocampus in the context of PTSD is to form new memories and guide behavior by comparing new sensory input to stored representations (Finsterwald et al., 2015). Thus, our aim was to investigate the effect of a WD in a modified rat model of PTSD that mimics both the cognitive and emotional features of the disorder simultaneously by using well validated behavioral paradigms (Bertoglio et al., 2006; Schneider et al., 2011). More importantly, since adolescence is a critical period in the neurobiological development of lifelong stress responses, another aim of this study was to use a longitudinal design that evokes measurable long-term fear memories associated with psychological trauma exposure. We report that consumption of a WD during adolescence results in increased behavioral manifestations of anxiety-like behaviors following exposure to stress. Our data reveal unique and highly distinctive neuroanatomical and molecular signatures linking WD consumption with vulnerability to psychological trauma.

## Materials and Methods

Experimental procedures were performed in compliance with the Loma Linda University School of Medicine regulations and institutional guidelines consistent with the National Institutes of Health *Guide for the Care and Use of Laboratory Animals*.

### Animals

Eighty adolescent male Lewis rats [postnatal day 21 (P21); LEW/Crl rats; RRID: RGD_737932] were acquired from Charles River Laboratories. Lewis rats are particularly prone to stress reactions, displaying many features in the stress neurocircuitry that are similar to those of children and adolescents exposed to traumatic stress and PTSD (i.e., poor HPA axis response to stress and proinflammatory milieu). Further, this rat strain has been extensively used to model PTSD (Cohen et al., 2006b), hippocampal alterations during traumatic stress (Goswami et al., 2012), and high-fat diet-induced obesity (Schmiedt et al., 2011). Thus, Lewis rats provide a powerful tool for understanding the interactions between genes and environment that underlie the development of PTSD-like behaviors. Upon arrival, rats were housed two per cage and maintained in conventional housing conditions (temperature, 21 ± 2°C; relative humidity, 45%; 12 h light/dark cycle with lights on at 7:00 A.M.). Animals had *ad libitum* access to food and water. Food consumption was recorded daily for the complete duration of the study. Body weights were recorded once a week.

### Study design

Rats were allowed to habituate to the housing conditions for 1 week. Following this acclimation period, the animals were randomly divided into the following two groups: one group received the control diet (CD; *n* = 44); while the other group received the WD (*n* = 36). Following 8 weeks receiving the diets, animals were further subdivided into two groups based on exposure to predator odor stress [PS; PS exposed, *n* = 48; CD unexposed (CDU), *n* = 32]. Animals were handled for 5 d before behavioral testing. Behaviors were evaluated at 4 and 8 weeks after special feeding commenced. This contextual pre-exposure paradigm has been validated and offers a novel way of separating the contextual and emotional memory components of PTSD-like behaviors (File, 1993; Treit et al., 1993; Fernandes and File, 1996; Bertoglio and Carobrez, 2000; Carobrez and Bertoglio, 2005; Bertoglio et al., 2006; Pawlak et al., 2008; Stern et al., 2010; Schneider et al., 2011; Gianlorenço et al., 2012; Chegini et al., 2014). In other words, novelty was used as a mild stressor and to create a context, whereas retesting measured emotionality and memory. Importantly, this paradigm is highly sensitive to pharmacological manipulations and lesions of the hippocampus, our region of interest (Kjelstrup et al., 2002). Only one behavioral test was performed per day to minimize carryover effects, increasing the behavioral stressor level intensity each day (from acoustic reflexes to forced swimming). All behaviors were recorded between 10:00 A.M. and 2:00 P.M. (the typical rat corticosterone nadir). Animals were killed after the completion of behavioral testing. The study followed a sequential experimental design. The number of rats in each experimental group was not evenly distributed among the different cohorts.

### Diets

The control diet (7 g% fat; catalog #F06405, Bio-Serv) and the Western high-fat diet (20 g% fat; catalog #F6724, Bio-Serv) are based on standard AIN-93G formulations. The diets were balanced for protein as a percentage of energy intake and for essential vitamins and minerals. In addition to the anhydrous milk fat used to prepare the WD, both diets contained soybean oil. We selected soybean oil as the fat source because this oil is one of the most common fat sources in the standard American diet. The WD contained 4.7 kcal/g, and the CD, 3.8 kcal/g. The WD contained a greater amount of cholesterol (0.2 g%) than the CD (0.036 g%). The diet pellets were analyzed using mass spectrometry to determine the fatty acid composition. We intended to mimic typical fat sources and compositions, increasing the translational and clinical relevance of our findings by using these diets.

### Predator odor stress

Experimental rats were exposed to soiled cat litter from a domestic male cat. To replicate previous studies, we used Fresh Step Unscented Multi-Cat Litter from Clorox Pet Products Company. The cat litter was in use by the cat for 2 d and sifted for stools (Cohen and Zohar, 2004; Cohen et al., 2006a; Goswami et al., 2010). The rats were placed in a standard plastic rat cage containing the soiled cat litter for 10 min. This model has ecological validity in that it mimics intense threatening psychological stress, with lasting consequences in cognitive and emotional processing (Adamec et al., 2006; Clay et al., 2011; Cohen et al., 2012). Control animals were exposed to fresh, unused litter for the same amount of time under otherwise equivalent conditions.

### Acoustic startle reflex and prepulse inhibition

The acoustic startle reflex (ASR) and prepulse inhibition (PPI) of the ASR paradigms (Geyer and Swerdlow, 2001) were executed using SR-Lab Acoustic Chambers (San Diego Instruments). All presented acoustic stimuli consisted of white noise. The background noise level was kept at 68 dB. The duration of acoustic stimuli was 20 ms for startle tones and 40 ms for prepulse tones. Trials were presented in a randomized interstimulus interval of 10–25 s. An experimental session took ∼20 min and started with a habituation program, consisting of 5 min of background noise. Following habituation, the initial block consisting of 11 120 dB acoustic startle trials was recorded. The second block consisted of 36 trials presented in a pseudorandomized order to evaluate the PPI of the ASR. In this second block, the trials included pulse-alone trials (120 dB) and PPI trials (five trials for each sound pressure level: 71, 74, or 80 dB). Finally, the third block of the session consisted of five additional pulse-alone startle trials and was used to determine short-term habituation of the ASR. After each session, the animals were returned to their cages and each chamber was cleaned with soap and water and thoroughly dried. Max startle amplitudes were averaged for each block. Based on empirical data and published studies, startle responses exhibited 52 ms after the presentation of the eliciting stimulus were filtered out from the analyses. The PPI of the ASR was calculated for each PPI trial as the percentage reduction of the average pulse-alone startle amplitude using the following formula:

(1) %PPI=100×[pulse alone−(prepulse+pulse)]/pulse alone.

### Open field test

The open field test (OFT) is normally used to evaluate emotionality based on the conflict rodents experience between the innate fear of open places and the drive to explore new areas (Hall, 1934, 1936). The dimensions of the acrylic open field maze were 43.2 cm (length) × 43.2 cm (width) × 30.5 cm (height). Animals were placed in the center of the field and recorded for 5 min using Ethovision version XT (Noldus Information Technology; RRID: SCR_000441, RRID: SCR_004074). Behaviors were recorded at normal room lighting conditions (269 lux illumination). The field was divided into a nine 14.4 cm^2^ zones using the software settings, allowing for the evaluation of inner and outer arena exploration. The center zone started ∼15 cm from each chamber wall. The chamber was cleaned with 70% ethanol between trials. The following equation was used to calculate the anxiety index:

(2) Anxiety index=1−[([center cumulative duration/total test duration]+[center total entries/total number of entries to center+corners])/2].

### Elevated plus maze

The elevated plus maze (EPM) evaluates emotionality on the same conflict paradigm as in the OFT (Pellow et al., 1985). Avoidance of the open arms is considered a measure of anxiety. The near infrared (NIR)-backlit EPM apparatus (catalog #ENV-564A, MedAssociates) consisted of two opposite open arms (50.8 × 10.2 cm) and two enclosed arms (50.8 × 10.2 × 40.6 cm) that were elevated 72.4 cm above the floor. The junction area between the four arms measured 10 × 10 cm. A raised edge (0.5 cm) on the open arms provided additional grip for the rats. Behaviors were recorded in a completely dark room. This NIR maze is specifically designed for video-tracking applications and eliminates variables associated with shadow/lighting. At the beginning of each 5 min trial, the rat was placed on the central platform facing an open arm. The apparatus was thoroughly cleaned after each test session. Behaviors were monitored via a monochrome video camera equipped with a NIR filer fixed above the EPM. In addition to the standard measures, we included extra measures derived from ethological analysis of behavior. The time spent on both types of arms, the number of entries into both types of arms, the latency to the first entry into any of the open arms, head-dipping zone frequency, and stretch attend postures (SAPs) were determined using Ethovision XT tracking software (Noldus Information Technology; RRID: SCR_000441, RRID: SCR_004074). From these data, we calculated the anxiety index according to the studies by Cohen et al. (2012) and Contreras et al., (2014):

(3) Anxiety index=1−[([OA cumulative duration/total test duration]+[OA entries/total number of entries to CA+OA])/2].

OA denotes Open Arms while CA represents Closed Arms in the elevated plus maze.

### Forced swim test

The forced swim test (FST) is based on the observation that rats, when exposed to an inescapable cylinder filled with water, will adopt a characteristic immobile posture in time (Porsolt et al., 1978; Abel, 1994). This adaptive behavior provides valuable information on emotionality and stress-coping strategies. The Plexiglas cylinder (height, 49.5 cm; diameter, 40 cm) was filled with 21 ± 2°C tap water to a height of 27 ± 5 cm. After the 15 min training session, the rat was dried with a towel, placed under heating lamps, and, subsequently, placed back into the home cage. Mobility was evaluated during the 5 min testing session (24 h after training) using Ethovision XT (Noldus Information Technology; RRID: SCR_004074). The time spent exhibiting high mobility (inversely related to depression and associated with anxiety) and the time spent immobile (positively related to depression) were calculated.

### Immunodetection

#### Western blot

Animals were killed with intraperitoneal administration of Euthasol (Virbac) and perfused transcardially with PBS. Animals were rapidly decapitated, and the brains were isolated and placed into an Alto stainless steel adult rat brain matrix (Stoelting). Unilateral (left hemisphere) micropunches were obtained from the hippocampus, medial prefrontal cortex, and amygdala. Micropunched tissue was immediately mixed with lysis buffer, gently sonicated, and stored at −80**°**C until the day of the experiment. The right brain hemisphere was isolated and snap frozen in liquid nitrogen. This region was used to optimize the Western blot conditions, and served as an internal control to determine the global effects of stress and diet on the FKBP51 protein levels. The tissue was pulverized with liquid nitrogen, homogenized, and stored at −80^°^C. For all experiments, a ratio of 1 mg of tissue to 8 μl of lysis buffer was used. The lysis buffer was composed of 4 ml of 0.5 m Tris-HCl, pH 6.8, 8 ml of 10% SDS, 6 ml of double-distilled H_2_O, and 2 ml of glycerol. After gentle sonication, the samples were centrifuged at room temperature for 10 min at 12,000 rpm. NuPAGE antioxidant (500 μl/gel) was used before loading protein samples. The proteins (15 μl/lane) were separated on NuPAGE 4-12% Bis-Tris gels (Invitrogen) and transferred for 7 min using iBlot Gel Transfer Stacks Nitrocellulose membranes (Invitrogen). After blocking for 1 h with Odyssey Blocking buffer (LI-COR Biosciences), the membranes were incubated overnight at 4**°**C in a 1× PBS 0.05% Tween-20 solution containing the anti-FKBP51 antibody (1:1000; catalog #PA1-020, ThermoFisher Scientific; RRID: AB_2103140). The membranes were then washed three times (10 min/wash) with 1× PBS 0.05% Tween-20, and incubated for 1 h with a goat anti-rabbit antibody (1:50,000; catalog #926-32211, LI-COR Biosciences; RRID: AB_621843), followed by three washes with 1× PBS. Finally, the membranes were incubated for 1 h with anti-β-actin (1:5000; catalog #Superclass, Sigma-Aldrich; RRID: AB_10013287) in 1× PBS 0.05% Tween-20. Subsequently, the membranes were washed three times with 0.05% Tween-20 in PBS and incubated for 1 h in goat anti-mouse (1:50,000; catalog #926-32220, LI-COR Biosciences; RRID: AB_621840). The membranes were washed three times with 1× PBS before imaging. Infrared signals were detected and quantitated using a LICOR Odyssey Scanner (LI-COR Biosciences).

#### Immunofluorescence

To prepare tissue for immunoﬂuorescence analyses, animals were submitted to fast and humane euthanasia with Euthasol and perfused transcardially with PBS, followed by 4% paraformaldehyde (PFA) in 0.1 m of phosphate buffer. Five hours after perfusion, the brain was carefully dissected and postﬁxed overnight in 4% PFA. Brains were shipped to NeuroScience Associates for cryoprotection, embedding, and Multi-Brain block processing (NeuroScience Associates). Multibrain coronal sections were dried at room temperature for 10–15 min, washed with PBS, and postﬁxed with 4% PFA for 10 min. Immunolabeling was performed with anti-FKBP51 antibody (1:250; catalog #ab2901, Abcam; RRID: AB_2103135) antibody or a mixture of anti-FKBP51 and Glut-1 antibody (1:1000; catalog #ab40084, Abcam; RRID: AB_2190927) to examine the colocalization of FKBP51 in endothelial cells. Antibody solutions were applied to the sections overnight at 4**°**C. On the following day, the Multi-Brain coronal sections were incubated with Alexa Fluor 488-conjugated donkey anti-rabbit (1:750; catalog #R37114, ThermoFisher Scientific; RRID: AB_2556542) and Alexa Fluor 594-conjugated donkey anti-mouse (1:750; Invitrogen; catalog #R37115, ThermoFisher Scientific; AB_2556543) antibodies. The Multi-Brain block sections were then washed and mounted with ProLong Gold Antifade containing DAPI (catalog #P36934, ThermoFisher Scientific). Primary antibody omission controls and antigen preabsorption controls were used to further conﬁrm the speciﬁcity of the immunoﬂuorescence. Sections were examined under a ﬂuorescence microscope (BZ9000; Keyence Corporation). Images were analyzed using the BZ-II Analyzer and prepared for publication with Photoshop CS6 software (Adobe; RRID: SCR_014199). DyLight 594-labeled *Lycopersicon esculentum* (tomato) Lectin (t-lectin; 1:200; catalog #DL-1177, Vector Laboratories; RRID: AB_2336416) was used as a marker of blood vessels. T-lectin was incubated overnight at 4**°**C in 0.25% bovine serum albumin with 0.25% Triton X-100 made in PBS, pH 7.4. For analyses, the threshold and morphologic user-deﬁned parameters were selected to maximize visualization of microglia or blood vessel-positive staining in the hippocampus. These parameters were kept consistent for all animals during image acquisition (*n* = 4 rats per group). Importantly, the Multi-Brain block preparation allowed for mass processing of 16 brains in a single unit (16 brain sections per slide). This provided consistent and uniform staining across sections and high-quality processing. All the images were acquired in *z*-stack mode (pitch = 0.5 μm; ∼15 images/stack). Images were taken from the dorsal (10 images) and ventral hippocampus (10 images) in each brain section using a 20× objective (numerical aperture, 0.75; WD 1.00) and a 3× digital zoom (total magnification, 60×; 20 images/brain section; ∼300 total images/brain; four brains/group). The BZ Analyzer Full Focus algorithm was run for each stack before analyzing each section. For automated unbiased quantification, the BZ-II Analyzer software Macro Hybrid Cell Count tool was used to calculate the area of positive staining, brightness, *R* integration, particle size, and blood vessel feret diameter in each image.

### Intraperitoneal glucose tolerance test

Rats were fasted for 12 h prior to receiving intraperitoneal glucose injections (2 g/kg). Blood glucose levels were measured in anesthetized rats by cutting the tail tip at 0, 30, 60, 90, and 120 min intervals. Glucose levels were measured using a glucometer (OneTouch UltraMini, LifeScan).

### Measuring leptin, triglycerides, and corticosterone concentrations using ELISA

Plasma leptin levels were measured using a commercial ELISA kit (catalog #ab100773, Abcam). Plasma samples were diluted with kit assay buffer (1:3 dilutions) following manufacturer instructions. ELISA plates were read at 450 nm on a plate reader (Molecular Devices). Specific concentrations for each sample were determined as mean absorbance using a standard curve of samples ranging from 0 to 8000 pg/ml. Values were then calculated and reported as picograms of leptin per milliliter.

In order to determine triglyceride concentrations, 3 ml of blood were collected transcardially during perfusion and added to tubes with 3 ml of 0.5 m EDTA. Blood samples were centrifuged at 1000 rpm, and plasma samples were extracted and placed in new tubes and frozen at −80°C until extraction. Samples were then diluted with an assay buffer kit (1:6 and 1:45 dilutions). Triglyceride concentrations were determined using the Triglyceride Quantification Assay following manufacturer instructions (Abcam). ELISA plates were read at 570 nm on a plate reader (Molecular Devices). Specific concentrations for each sample were determined as mean absorbance using a standard curve of samples ranging from 0 to 1000 pmol. Values were then calculated and reported as the number of milligrams of triglyceride per deciliter.

In order to determine corticosterone concentrations noninvasively, fecal samples were collected at 1 week after the psychological stressor. Samples were frozen at −80°C until extraction. Extraction followed previous methods (Turner et al., 2012). Samples were defrosted, weighed, and pulverized. Ethanol was then added to these samples, rotated overnight, vortexed, and centrifuged, and the resulting supernatant was extracted. Samples were then diluted with kit assay buffer (1:4 dilutions). Corticosterone concentration was determined using the Correlate-EIA Kit (Assay Designs) following manufacturer instructions. ELISA plates were read at 405 nm on a plate reader (μQuant, BioTek Instruments). Specific concentrations for each sample were determined as the percentage bound using a standard curve of samples ranging from 32 to 20,000 pg/ml. Values were then calculated and reported as picograms of corticosterone per milliliter.

### Magnetic resonance imaging

*Ex vivo* brains underwent anatomical high-resolution T2-weighted anatomical magnetic resonance imaging (MRI) using an 11.7 T Avance scanner (BioSpin, Bruker) The multiecho sequence had the following acquisition parameters: matrix, 256 × 256 matrix; 25 slices covering the whole brain at 0.6 mm slice thickness; field of view, 2 cm; repetition time/echo, 2903/10.2 ms; 10 echos; two averages for a scan time of 25 min. This resulted in a 78 × 78 × 600 μm/pixel resolution to enhance total volumetric hippocampal and ventricular analysis.

Volumetric analysis of total brain and hippocampi and lateral and third ventricles was performed on coronal slices using Cheshire image-processing software (Hayden Image/Processing Group) by investigators who were unaware of treatment groups. Regions of interest were manually delineated on each slice, and included whole-brain volumes, left and right hippocampi, and the lateral and third ventricles spanning the entire rostrocaudal extent using anatomically defined landmarks. The hippocampi were defined to include the first appearance of dorsal hippocampal regions (i.e., dentate gyrus −1.80 mm from bregma) and continued until hippocampus merges into the subiculum (−7.04 mm from bregma). Automated whole-brain volumes were determined starting from where the olfactory bulbs start to merge with the cortical gray matter (3.70 mm from bregma) to the last instance of the cortical mantel prior to the cerebellum (−9.16 mm from bregma). The ventricular system was defined as the left and right lateral ventricles at their first appearance adjacent to the dorsal hippocampi (−4.52 mm from bregma) and spanned its entirety ending where the nucleus accumbens recedes from view (2.70 mm from bregma). The third ventricle was collected just prior to where the corpus callosum begins to merge (−5.60 mm from bregma) and continues until the optic chiasm is no longer visible (−0.40 mm from bregma). All data were extracted and summarized in Excel. To account for variability between individual animals, the first and last hippocampal slice data were excluded from the final analysis.

### Statistical analysis

We analyzed the data using SPSS version 23 (IBM) and GraphPad Prism versions 6g and 7.0 (GraphPad; RRID: SCR_002798) via a two-way ANOVA (TW-ANOVA), multivariate ANOVA, or multivariate analysis of covariance, and multiple regression analysis. We used Tukey’s HSD test to assess significant *post hoc* differences between groups. We also used the Kolmogorov–Smirnov and Shapiro–Wilk normality tests together with the Grubbs’ method, also known as extreme studentized deviate (www.graphpad.com), to investigate outliers and spread. We used one-tailed Pearson’s correlation tests to test the hypothesis that metabolic alterations are associated with stress reactivity and brain volumes. For all of the other data, we analyzed differences among groups using Student’s *t* test. We considered differences significant if *p* < 0.05. The data are shown as the mean ± SEM.

## Results

### Caloric intake, body weight, and glucose metabolism are altered by the Western high-fat diet

By using well defined and typical fat sources and fractions in our diets, we intended to increase the translational and clinical relevance of our findings (Table 1). We found that rats exposed to the WD showed increased caloric intake following stress when compared with the rats that consumed the CD at P65 (*p* < 0.05). This effect was sustained until the completion of the study. Both PS exposure and WD consumption increased caloric consumption at 11 weeks (PS: *F*_(1,10)_ = 18.96, *p* = 0.0014; diet: *F*_(1,10)_ = 13.99, *p* = 0.0038; interaction: *F*_(1,10)_ = 0.086, *p* = 0.77). Similarly, we found a significant main effect of the PS exposure and the WD on increasing the body weight of rats at 11 weeks (PS: *F*_(1,24)_ = 25.12 *p* < 0.0001; diet: *F*_(1,24)_ = 25.81, *p* < 0.0001; interaction: *F*_(1,24)_ = 0.71, *p* = 0.41). Table 2 summarizes the effects of the diet on body weight and consumption.

**Table 1:** Detailed compositional analysis of the research diets

	CD	WD
Carbohydrates (% kcal)	64.7	43.1
Protein (% kcal)	18.8	15.5
Fat (% kcal)	16.5	41.4
Total kcal	3.77	4.57
Fatty acids (g/100 g)		
C4:0	ND	0.68
C6:0	ND	0.44
C8:0	ND	0.29
C10:0	ND	0.43
C12:0	ND	0.59
C14:0	ND	2.10
C16:0	0.81	5.59
C18:0	0.32	2.71
Total saturated	1.13	12.83
C16:1	0.04	0.42
C18:1	1.57	5.50
Total monosaturated	1.61	5.92
C18:2n6	3.55	1.04
C18:3n3	0.48	0.36
C20:4n6	ND	0.10
C20:5n3	ND	ND
C22:5n3	ND	ND
C22:6n3	ND	ND
Total polyunsaturated	4.09	1.50

The level of dietary fat was ∼7% of dry weight in the CD or 20% in the WD. Gas chromatography coupled with mass spectrometry revealed high levels of saturated fatty acids (11-fold difference) and monosaturated fatty acids (4-fold difference) in the WD when compared with the CD. The CD contained higher levels of omega-6 polyunsaturated fatty acids. ND, Not determined.

**Table 2:** WD and PS alter major biometric parameters

Biometric parameter	Group				*p* Value		
	CDU	CDE	WDU	WDE	Diet	PS	Interaction
Body weight (g)	429 ± 7.6	461 ± 3.6	462 ± 13.6	506 ± 4.3*,***	**<0.0001**	**<0.0001**	0.409
Food consumption (kcal/d)	72 ± 5.4	84 ± 3.8	84 ± 1.3	98 ± 1.4*,***	**0.002**	**0.001**	0.749
Corticosterone (ng/0.5 g feces)	8.2 ± 1.7	7.6 ± 1.5	6.8 ± 0.7	7.1 ± 0.5	0.206	0.827	0.505
FBG (mg/dl)	137 ± 4.7	134 ± 7.5	165 ± 9.2	137 ± 7.8	0.087	0.086	0.170
PBG (mg/dl)	127 ± 9.4	167 ± 8.8****	129 ± 5.1	109 ± 4.7***	**0.002**	0.199	**0.001**
Plasma leptin (pg/ml)	612 ± 82.8	603 ± 163.7	1211 ± 257.6	1553 ± 282.2***	**0.006**	0.481	0.459
Plasma triglycerides (mg/dl)	626 ± 18.1	581 ± 37.7	679 ± 35.7	643 ± 38.9	0.116	0.252	0.900

The rats that consumed the WD showed significant alterations in body weight and food consumption when compared with animals consuming the CD (*n* = 12–16 rats/group). Exposure to traumatic stress (*F*_(1,24)_ = 25.12; *p* < 0.0001) and WD consumption (*F*_(1,24)_ = 25.81, *p* < 0.0001) increased body weights. No interactions were observed between traumatic stress and diet (*F*_(1,24)_ = 0.71, *p* = 0.40). Notably, we found that the rats that consumed the WD showed a significant increase in caloric intake (PS: *F*_(1,10)_ = 18.96, *p* = 0.0014; diet: *F*_(1,10)_ = 13.99, *p* = 0.0038; PS × diet interaction: *F*_(1,10)_ = 0.086, *p* = 0.78; *n* = at least 6 cages/group; 2 rats per cage). We found no significant differences in FBG levels between the diet and stress groups (PS: *F*_(1,24)_ = 3.20, *p* = 0.086; diet: *F*_(1,24)_ = 3.18, *p* = 0.087; interaction: *F*_(1,24)_ = 2.01, *p* = 0.17). However, we found significant main effect interactions in PBG levels (PS: *F*_(1,24)_ = 0.0016, *p* = 0.97; diet: *F*_(1,24)_ = 2.03, *p* = 0.17; interaction: *F*_(1,24)_ = 11.66, *p* = 0.0023; *n* = 12–16 rats/group). The WD and PS did not alter corticosterone (CORT) levels (diet effect: *F*_(1,24)_ = 1.69, *p* = 0.21; PS: *F*_(1,24)_ = 0.049, *p* = 0.0.83); interaction: *F*_(1,24)_ = 0.46, *p* = 0.51; *n* = 12-13 rats/group). Values are presented as the mean ± SEM. The diet type had a significant effect on plasma leptin levels (diet: *F*_(1,11)_ = 11.53, *p* = 0.0060; PS: *F*_(1,11)_ = 0.53, *p* = 0.48; interaction *F*_(1,11)_ = 0.59, *p* = 0.46). *Post hoc* testing revealed that WDE rats had significantly higher plasma leptin levels when compared with those of CDE rats (*p* = 0.0456). Interestingly, plasma triglyceride levels were not affected by the diet and stress exposure (diet: *F*_(1,12)_ = 2.87, *p* = 0.12; PS: *F*_(1,12)_ = 1.45, *p* = 0.25; interaction *F*_(1,12)_ = 0.016, *p* = 0.90). Bold denotes statistically significant effects.

*p < 0.05.

***p < 0.001.

****p < 0.0001.

Fasting blood glucose (FBG) levels were similar between dietary groups at the end of the study (PS: *F*_(1,24)_ = 3.199, *p* = 0.086; diet: *F*_(1,24)_ = 3.18, *p* = 0.087; interaction: *F*_(1,24)_ = 2.01, *p* = 0.17). We found that the effects of PS exposure on glucose metabolism differed between the diet groups, as revealed by statistically significant interaction effects on postprandial blood glucose (PBG) levels at 2 h following intraperitoneal glucose injection (PS: *F*_(1,24)_ = 0.0016, *p* = 0.97; diet effect *F*_(1,24)_ = 2.03, *p* = 0.17; interaction *F*_(1,24)_ = 11.66, *p* = 0.0023). *Post hoc* identified the source of this interaction, which revealed reduced PBG levels in the WD rats that were exposed to PS (WDE) when compared with control rats exposed to PS (CDE) at 2 h following intraperitoneal glucose administration (*p* = 0.0025). Group comparisons confirmed that the rats in the WD group exhibited alterations in glucose metabolism, as evidenced by differences between fasting blood glucose and 2 h postprandial glucose levels (*p* < 0.001).

We measured the levels of corticosterone (CORT) at 1 week after traumatic stress exposure (P95). Although we observed a trend for higher CORT levels in the rats that consumed the WD, TW-ANOVA revealed no significant main effects (PS: *F*_(1,24)_ = 0.049, *p* = 0.0.83; diet: *F*_(1,24)_ = 1.69, *p* = 0.206; interaction: *F*_(1,24)_ = 0.459, *p* = 0.505).

As anticipated, we found that the obesogenic diet had a significant effect on plasma leptin levels (diet: *F*_(1,11)_ = 11.53, *p* = 0.0060; PS: *F*_(1,11)_ = 0.53, *p* = 0.48; interaction *F*_(1,11)_ = 0.59, *p* = 0.46). The source of this interaction was identified *post hoc*, which revealed increased leptin levels in the WDE rats when compared with CDE rats (*p* = 0.0456). Interestingly, we found no significant main effects on plasma triglyceride levels (diet: *F*_(1,12)_ = 2.87, *p* = 0.12; PS: *F*_(1,12)_ = 1.45, *p* = 0.25; interaction *F*_(1,12)_ = 0.016, *p* = 0.90). Table 2 summarizes the metabolic alterations caused by the diet, PS exposure, and their main effect interactions.

### Experimental design directly addresses emotional memory and anxiety-related behaviors

**Figure 1.**
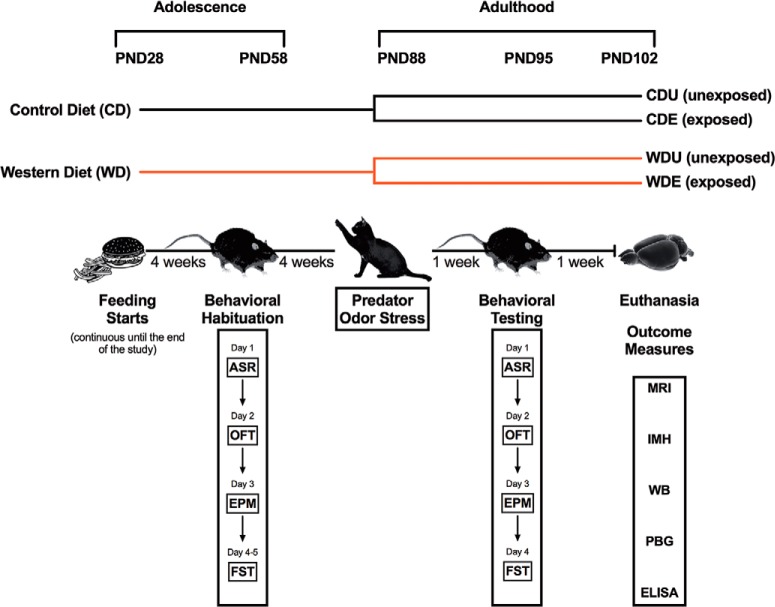
Timeline of experimental procedures, behavioral tests, and outcome measures. Abbreviations: PND, postnatal day; CDU, control diet unexposed; CDE, control diet exposed; WDU, Western high-fat diet unexposed; WDE, Western high-fat diet exposed; ASR, acoustic startle reflex; OFT, open field test; EPM, elevated plus maze; FST, forced swim test; MRI, magnetic resonance imaging; IMH, immunohistochemistry; WB, Western blot; PBG, postprandial blood glucose; ELISA, enzyme-linked immunosorbent assay.

Following 4 weeks of receiving the diets, we evaluated the rats for unconditioned behavioral responses at P58 to P65. Overall, we found that both groups exhibited similar unconditioned behaviors (*p* > 0.05). Table 3 summarizes the main behavioral outcomes during late adolescence. The rats were exposed to a predator odor model of PTSD at P88. We assessed traumatic stress responses and emotional memory at 1 week following predatory odor trauma (P95). As stated earlier, the usefulness of this validated habituation-test approach rests on the fact that it facilitates the understanding of shifts in emotional states from unconditional (habituation and novelty phase) to a hippocampus-driven learned form of fear response (testing phase; Bertoglio and Carobrez, 2000; Bertoglio et al., 2006). The experimental timeline and design are summarized in Figure 1.

**Table 3:** Behavioral outcomes during habituation

Behavior	CD	WD	*p* Value
ASR (vMax, mV)	346.90 ± 66.0	267.60 ± 40.0	n.s.
PPI (%)	67.98 ± 3.4	72.89 ± 5.0	n.s.
EPM-entries (frequency)	5.17 ± 1.8	7.63 ± 1.8	n.s.
EPM-anxiety index (index from 0 to 1)	0.47 ± 0.0	0.56 ± 0.1	n.s.
FST-high mobility (time duration, %)	10.88 ± 1.6	12.34 ± 1.3	n.s.

Values are presented as the mean ± SEM. Baseline ASR, PPI of the ASR, and EPM and FST indices of anxiety were evaluated at P58 (4 weeks after WD consumption started and 4 weeks before traumatic stress). No significant differences were observed between diet groups (*p* > 0.05, *n* = 8–23 rats per group/behavioral outcome). n.s., Not significant.

### Diet groups exhibited similar avoidance behaviors to an short-term predator odor stress model of PTSD

**Figure 2.**
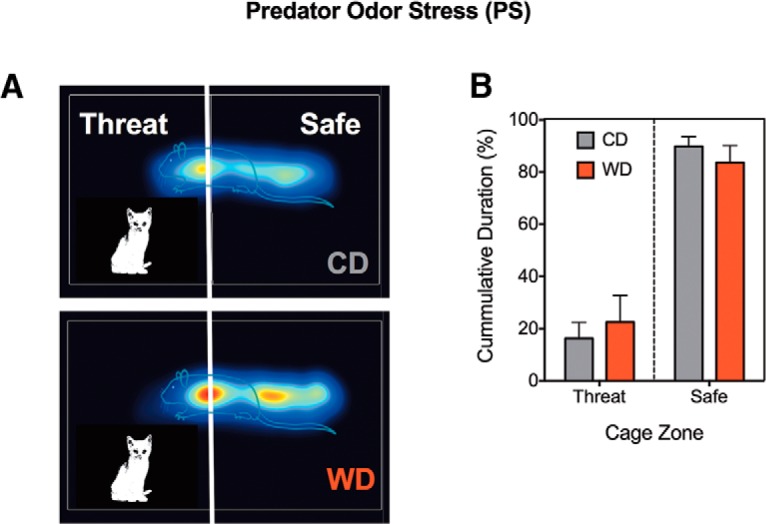
Exposure to PS evokes short-term avoidance behaviors. ***A***, Heat maps depicting the time spent in the threat zone (area of the cage with cat urine odor) and safe zone during PS exposure at P88. ***B***, Analyses showed marked differences in approach–avoidance behaviors. Rats exhibited a strong preference for the safe zone (*F*_(1,32)_ = 78.87, *p* < 0.0001). The diet type did not alter this behavior (*F*_(1,32)_ = 1.43 × 10^−10^, *p* = 1.00). No interactions were found between the time spent in the odor threat zone and diet type consumed (*F*_(1,32)_ = 0.68, *p* = 0.42). All data are presented as the mean ± SEM; *n* = 8-10 rats/group.

Exposing rats to a well established PS model of PTSD elicited marked alterations in stress-coping strategies (Fig. 2). CDU animals were exposed to fresh, unused litter. During the 10 min PS exposure, we found that rats significantly avoided the odor threat zone, confirming the validity of this model to elicit stress-related behaviors (zone duration: *F*_(1,32)_ = 78.87, *p* < 0.0001). Both dietary groups exhibited similar approach–avoidance conflict responses to the odor threat (diet: *F*_(1,32)_ = 1.430 × 10^−10^, *p* = 1.00). No significant interaction effects were observed between the time spent in the odor threat zone and the diet consumed (*F*_(1,32)_ = 0.68, *p* = 0.42). Following PS exposure, we investigated the effects of PS, diet, and interactions between these factors on behavior, brain volumes, and key metabolic and stress biomarkers. Our 2 × 2 design included the following four groups: CDU, CDE, WD-unexposed (WDU), and WDE rats.

### Consumption of a WD impairs startle responses to acoustic stimuli following traumatic stress

**Figure 3.**
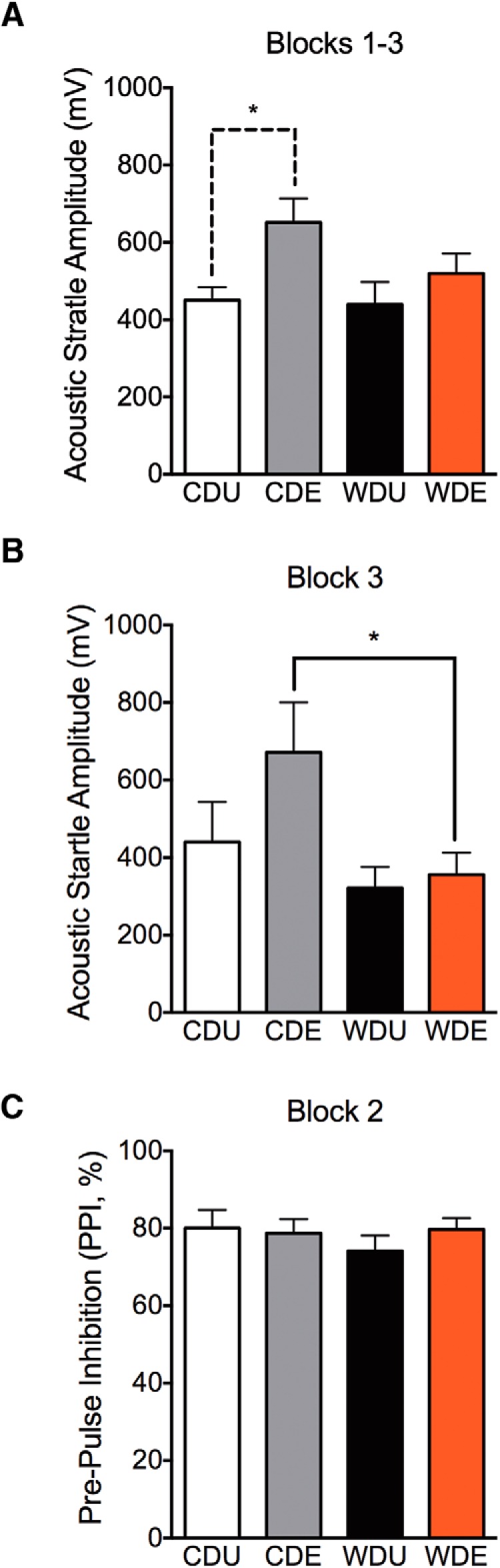
WD reduces traumatic stress-induced startle responses. ***A***, We found that the maximum ASR amplitude increased at 1 week post-PS (PS: *F*_(1,25)_ = 5.43, *p* = 0.028; diet: *F*_(1,25)_ = 1.41, *p* = 0.25; interactions: *F*_(1,25)_ = 1.00, *p* = 0.33). *Post hoc* testing revealed a significant increase in ASR amplitude in CDE rats when compared with controls (*p* < 0.05). ***B***, WDE rats exhibited reduced ASR responses during the last recording block when compared with CDE rats (diet: *F*_(1,23)_ = 7.28, *p* = 0.013; Tukey’s adjusted for WDE vs CDE, *p* = 0.028). PS exposure (*F*_(1,23)_ = 2.73, *p* = 0.11) or diet–PS exposure interactions (*F*_(1,23)_ = 1.43, *p* = 0.24) did not affect the short-term habituation of the ASR. ***C***, TW-ANOVA revealed no significant effect of diet (*F*_(1,26)_ = 0.35, *p* = 0.56), PS (*F*_(1,26)_ = 0.39, *p* = 0.54), or interaction between these factors (*F*_(1,26)_ = 0.87, *p* = 0.36) on the PPI of the ASR. Data presented are presented as the mean ± SEM. *Post hoc* test, *p < 0.05, **p < 0.01, *n* = 10–23 rats.

The major outcome measure of startle reactivity is the ASR magnitude, a direct measure of attention and arousal states. In agreement with several studies, we found that PS exposure increased the magnitude of the ASR (PS: *F*_(1,25)_ = 5.43, *p* = 0.028; Fig. 3*A*). This effect was significant in CDE rats when compared with CDU rats (*t*_(8)_ = 2.83, *p* = 0.022). This finding validates the reported effects of PS on the ASR amplitude. Analyses revealed no significant main effects of the diet or interactions on ASR magnitudes (diet: *F*_(1,25)_ = 1.41, *p* = 0.25; interaction: *F*_(1,25)_ = 1.00, *p* = 0.33). Interestingly, we found that the WD rats showed increased short-term habituation of the ASR, as evidenced by reduced startle responses in the third ASR block (*F*_(1,23)_ = 7.28, *p* = 0.013). This diet effect was significant between the rats that were exposed to PS (CDE vs WDE: *p* = 0.028; Fig. 3*B*). We found no significant main PS exposure or diet–PS exposure interaction on the magnitude of the ASR during the short-term habituation block (PS: *F*_(1,23)_ = 2.73, *p* = 0.11; interaction: *F*_(1,23)_ = 1.44, *p* = 0.24) . TW-ANOVA revealed no significant effect of the diet, traumatic stress, or interactions between these factors on the prepulse inhibition of the ASR (PS: *F*_(1,26) =_ 0.39, *p* = 0.54; diet: *F*_(1,26)_ = 0.35, *p* = 0.56; interaction: *F*_(1,26)_ = 0.87, *p* = 0.36; Fig. 3*C*).

### Consumption of a WD increases anxiety-like behaviors following exposure to traumatic stress

**Figure 4.**
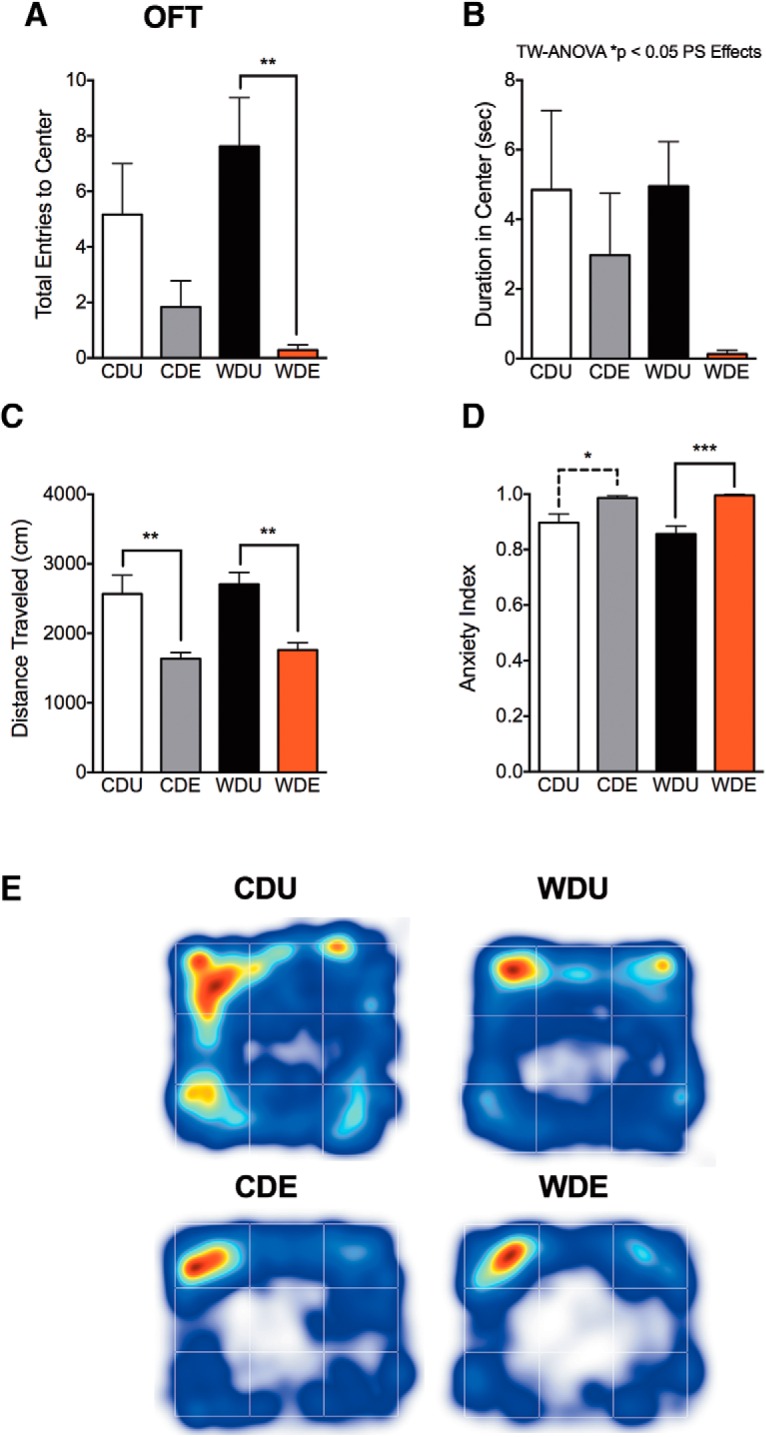
WD confers vulnerability to OFT anxiety-like behaviors following traumatic stress exposure. ***A***, PS exposure reduced the number of entries to the center of the OFT (PS: *F*_(1,23)_ = 14.48, *p* = 0.0009; diet: *F*_(1,23)_ = 0.11, *p* = 0.75; diet × PS interactions: *F*_(1,23)_ = 2.04, *p* = 0.17). *Post hoc* comparisons indicated that the effect of PS was particularly robust in WDE rats when compared with WDU controls (Tukey’s test adjusted, *p* = 0.0036). ***B***, ANOVA revealed that PS decreased the duration in the center (*F*_(1,23)_ = 5.02, *p* = 0.035), while diet (*F*_(1,23)_ = 0.83, *p* = 0.37) and diet–PS exposure interactions (*F*_(1,23)_ = 0.97, *p* = 0.34) showed no significant main effects. ***C***, PS exposure reduced the total distance traveled in the open field arena (*F*_(1,23)_ = 32.85, *p* = 0.0001). Analyses revealed no significant main effect of the diet (*F*_(1,23)_ = 0.37, *p* = 0.55) or diet–PS exposure interactions (*F*_(1,23)_ = 0.049, *p* = 0.83) on locomotor activity in the open field arena. ***D***, PS exposure increased indices of anxiety on the OFT (PS: *F*_(1,23)_ = 25.97, *p* < 0.0001). The main effects of the diet (*F*_(1,23)_ = 0.37, *p* = 0.55) and diet–PS exposure interactions (*F*_(1,23)_ = 1.35, *p* = 0.26) showed no statistical significance on the OFT anxiety index. ***E***, OFT heat map analyses show that thigmotaxis was particularly robust in the WDE group. All data are presented as the mean ± SEM. *p < 0.05, **p < 0.01, *n* = 6–10 rats/group.

Next, we examined whether the rats consuming the WD would exhibit anxiety-like behaviors following PS exposure. We assessed diet- and PS-induced behavioral changes in the OFT, in which rats exhibiting anxiety-like behaviors tend to avoid the center area of an open-field arena. As expected, we observed that exposure to PS had a significant effect on the number of visits to the center area (PS: *F*_(1,23)_ = 14.48, *p* = 0.0009). *Post hoc* comparisons indicated that this effect was particularly robust in WDE rats when compared with WDU controls (*p* = 0.0036; Fig. 4*A*). Diet or diet–PS exposure interactions did not affect the number of visits to the center area of the open field (diet: *F*_(1,23)_ = 0.11, *p* = 0.75; interaction: *F*_(1,23)_ = 2.04, *p* = 0.17). We found that PS significantly decreased the time spent (duration) in the center of the open-field arena (PS: *F*_(1,23)_ = 5.02, *p* = 0.035; Fig. 4*B*). The diet type did not affect the duration in the center of the open field (diet: *F*_(1,23)_ = 0.83, *p* = 0.37; interaction: *F*_(1,23)_ = 0.97, *p* = 0.34). Interestingly, we found that PS exposure reduced the distance traveled in the open field arena (PS: *F*_(1,23)_ = 32.85, *p* = 0.0001; Fig. 4*C*). There was not a significant main effect of the diet or diet–PS exposure interactions on locomotor activity in the open field arena (diet: *F*_(1,23)_ = 0.37, *p* = 0.55; interaction: *F*_(1,23)_ = 0.049, *p* = 0.83). The OFT anxiety index, which considers the time and frequency of entries to the borders and thus inherently corrects for locomotor activity, showed the sensitivity of the OFT to detect the anxiogenic effects of PS at 8 d postexposure (PS: *F*_(1,23)_ = 25.97, *p* < 0.0001; Fig. 4*D*). We did not find significant main effects of the diet or diet–PS exposure interactions on the OFT anxiety index (diet: *F*_(1,23)_ = 0.37, *p* = 0.55; interaction: *F*_(1,23)_ = 1.35, *p* = 0.26). Ethovision XT-generated heat maps show increased thigmotaxis in the rats exposed to the PS (Fig. 4*E*).

**Figure 5.**
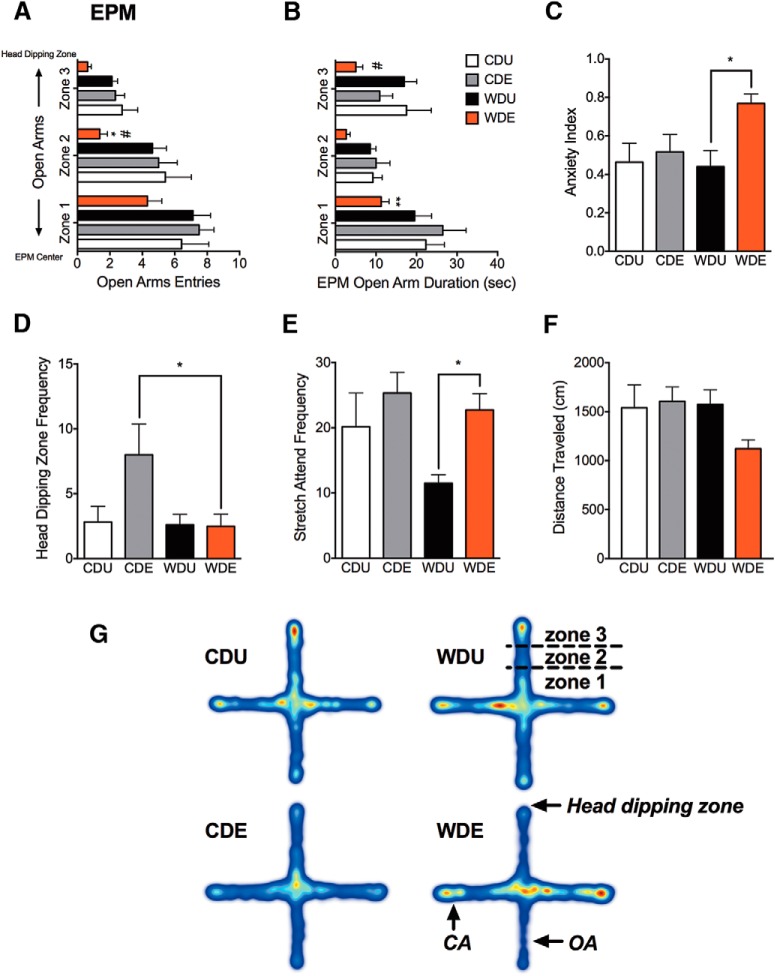
WD increases anxiety-like behaviors in the EPM following PS. Each open arm was subdivided into three zones (zone 1, proximal; zone 2, medial; zone 3, distal) to eliminate false-positive results in arm entry statistics. ***A***, The diet type significantly affected the number of entries to the medial section of the open arms (diet: *F*_(1,24)_ = 4.68, *p* = 0.041). WDE rats showed a reduced number of entries to zone 2 and zone 3 of the open arms when compared with CDE rats (*p* < 0.05). PS exposure (*F*_(1,24)_ = 3.23, *p* = 0.085) or diet–PS exposure interactions (*F*_(1,23)_ = 1.93, *p* = 0.18) did not affect the number of entries to the medial zone of the open arms. ***B***, Notably, we found a significant main diet effect on the time the rats spent in the proximal zones of the EPM open arms (for zone 1: *F*_(1,23)_ = 4.81, *p* = 0.039). *Post hoc* testing revealed that WDE rats spent less time in the proximal and medial parts of the open arms when compared with CDE rats (*p* < 0.05). PS exposure main effects (*F*_(1,23)_ = 0.25, *p* = 0.62) or diet–PS exposure interactions (*F*_(1,23)_ = 2.30, *p* = 0.14) showed no statistical significance. PS exposure decreased the time spent in the distal zones of the EPM open arms (for zone 3: *F*_(1,23)_ = 6.59, *p* = 0.017). Analyses revealed no significant diet (*F*_(1,23)_ = 0.80, *p* = 0.38) or diet–PS exposure interactions effects (*F*_(1,23)_ = 0.55, *p* = 0.47). ***C***, PS exposure had a significant effect on the EPM index of anxiety (*F*_(1,24)_ = 5.65, *p* = 0.026). *Post hoc* analyses revealed that the PS-exposed rats exhibited an increased anxiety index when compared with controls (WDE vs WDU, *p* = 0.022). Analyses revealed no significant main effects of the diet (*F*_(1,24)_ = 2.03, *p* = 0.17) or diet–PS exposure interactions (*F*_(1,24)_ = 2.94, *p* = 0.099) on the anxiety index. ***D***, We found that the diet type significantly affected the frequency of when the nose point of the rats was in the head-dipping areas (*F*_(1,24)_ = 4.61, *p* = 0.042). *Post hoc* comparisons indicated a decreased nose in head-dipping zone frequency in WDE rats when compared with the CDE rats (*p* < 0.05). PS exposure (*F*_(1,24)_ = 3.60, *p* = 0.070) and diet–PS exposure interactions (*F*_(1,24)_ = 3.96, *p* = 0.058) did not have a significant effect on head-dipping zone frequency. ***E***, PS exposure increased SAP frequency (PS: *F*_(1,24)_ = 7.17, *p* = 0.013). *Post hoc* testing showed increased SAPs in the WDE rats when compared with WDU rats (*p* < 0.05). The diet type (*F*_(1,24)_ = 3.37, *p* = 0.079) and diet–PS exposure interactions (*F*_(1,24)_ = 0.98, *p* = 0.33) did not show significant main effects on SAP frequency. ***F***, We found no significant main effect of diet (*F*_(1,24)_ = 0.94, *p* = 0.16), PS exposure main effect (*F*_(1,24)_ = 1.54, *p* = 0.23), or interaction between these factors (*F*_(1,24)_ = 2.76, *p* = 0.11) on the total distance traveled during EPM testing. ***G***, Heat maps show that WDE rats spent more time in the closed arms when compared with CDE rats. All data are presented as the mean ± SEM. *p < 0.05, **p < 0.01, *n* = 6–10 rats/group. For ***A*** and ***B***: *p < 0.05 or **p = 0.01 for CDE vs WDE; #*p* < 0.05 for WDU vs WDE.

Subsequently, we evaluated whether consumption of the WD alters emotionality after traumatic stress exposure using another classic test of anxiety-like behavior in rats, the EPM. Each open arm was subdivided into three distinctive zones using the tracking software. We found that the WD significantly reduced the number of entries to the middle section of the open arms (diet: *F*_(1,24)_ = 4.68, *p* = 0.041; Fig. 5*A*). *Post hoc* comparisons revealed a significantly reduced number of entries to medial (zone 2) and distal (zone 3) parts of the open arms in CDE and WDE rats (*p* < 0.05). We found significant differences in entries to the medial zone of the open arm when comparing WDE to CDE rats (*p* < 0.05). PS exposure did not affect the number of entries to the middle zone of the open arms (PS: *F*_(1,24)_ = 3.23, *p* = 0.085; no interactions: *F*_(1,23)_ = 1.93, *p* = 0.18). TW-ANOVA showed that the rats that consumed the WD spent less time in the proximal zones of the EPM open arms (diet effect on Zone 1: *F*_(1,23)_ = 4.81, *p* = 0.039; Fig. 5*B*). *Post hoc* testing revealed a significant reduction in the time spent in proximal and medial parts of the open arms in WDE rats when compared with CDE rats, supporting an anxiogenic effect of the WD following PS exposure (*p* < 0.05). We did not find significant PS exposure main effects or diet–PS exposure interactions (PS: *F*_(1,23)_ = 0.25, *p* = 0.62; interaction: *F*_(1,23)_ = 2.30, *p* = 0.14). Consistent with the effects of PS exposure on the time the rats spent in the center of the OFT arena, we found that exposure to traumatic psychological stress decreased the duration in the distal zones of the EPM open arms (diet effect on zone 3: *F*_(1,23)_ = 6.59, *p* = 0.017; Fig. 5*B*). We did not find significant main diet effects or diet–PS exposure interactions (diet: *F*_(1,23)_ = 0.80, *p* = 0.38; interaction: *F*_(1,23)_ = 0.55, *p* = 0.47). Our analyses revealed that PS exposure had a significant effect on the EPM anxiety index (PS: *F*_(1,24)_ = 5.65, *p* = 0.026; Fig. 5*C*). *Post hoc* analyses revealed that the WDE rats exhibited increased indices of anxiety when compared with WD controls (*p* = 0.022). We found no significant main effects of the diet or diet–PS exposure interactions on the EPM anxiety index (diet: *F*_(1,24)_ = 2.03, *p* = 0.17; interaction: *F*_(1,24)_ = 2.94, *p* = 0.099). Notably, we found that the rats that consumed the WD exhibited behavioral alterations associated with anxiety, including reduced nose-tracking frequency in the head-dipping zone (diet: *F*_(1,24)_ = 4.61, *p* = 0.042; Fig. 5*D*). Although it was apparent that PS increased the head-dipping zone frequency in CDE rats when compared with CDU rats, the comparison did not reach statistical significance. In contrast, *post hoc* comparisons indicated that WDE rats exhibited decreased visits to the head-dipping zone when compared with the CDE rats (*p* < 0.05). Analyses indicated no significant PS exposure effects or diet–PS exposure interactions on this ethological parameter (PS: *F*_(1,24)_ = 3.60, *p* = 0.070; interaction: *F*_(1,24)_ = 3.96, *p* = 0.058). TW-ANOVA demonstrated a significant main effect of PS exposure on increasing the number of SAPs (PS: *F*_(1,24)_ = 7.17, *p* = 0.013; Fig. 5*E*). *Post hoc* testing showed increased SAPs in the WDE rats when compared with WDU controls (*p* < 0.05), confirming the PS-induced anxiety-like behaviors in the rats that consumed the WD. The diet type or diet–PS exposure interactions did not have significant main effects on SAP frequency (diet: *F*_(1,24)_ = 3.37, *p* = 0.079; interaction: *F*_(1,24)_ = 0.98, *p* = 0.33). The anxiogenic effects of the diet and PS were not associated with the total distance traveled in the EPM (diet: *F*_(1,24)_ = 0.94, *p* = 0.16; PS: *F*_(1,24)_ = 1.54, *p* = 0.23; interaction: *F*_(1,24)_ = 2.76, *p* = 0.11; Fig. 5*F*). Heat maps show that WDE rats avoided open arm exploration and spent most of the time in the closed arms (Fig. 5*G*).

The impact of the WD and PS exposures were also tested in the FST in order to investigate whether depressive-like behaviors were also affected. Interestingly, we found no significant main effects of the diet, PS exposure, or interaction between these factors on the duration of high-mobility behaviors during the FST testing session (diet: *F*_(1,17)_ = 0.10, *p* = 0.753; PS: *F*_(1,17)_ = 1.76, *p* = 0.20; interaction: *F*_(1,17)_ = 0.00089, *p* = 0.98; data not shown). TW-ANOVA revealed no significant main diet effects, PS exposure effects, or significant interaction between these factors on depressive-like behaviors, as evaluated by the immobility duration during the FST (diet: *F*_(1,17)_ = 0.067, *p* = 0.80; PS: *F*_(1,17)_ = 1.26, *p* = 0.28; interaction: *F*_(1,17)_ = 0.062, *p* = 0.81; data not shown).

### The WD increases FKBP51 protein levels

**Figure 6.**
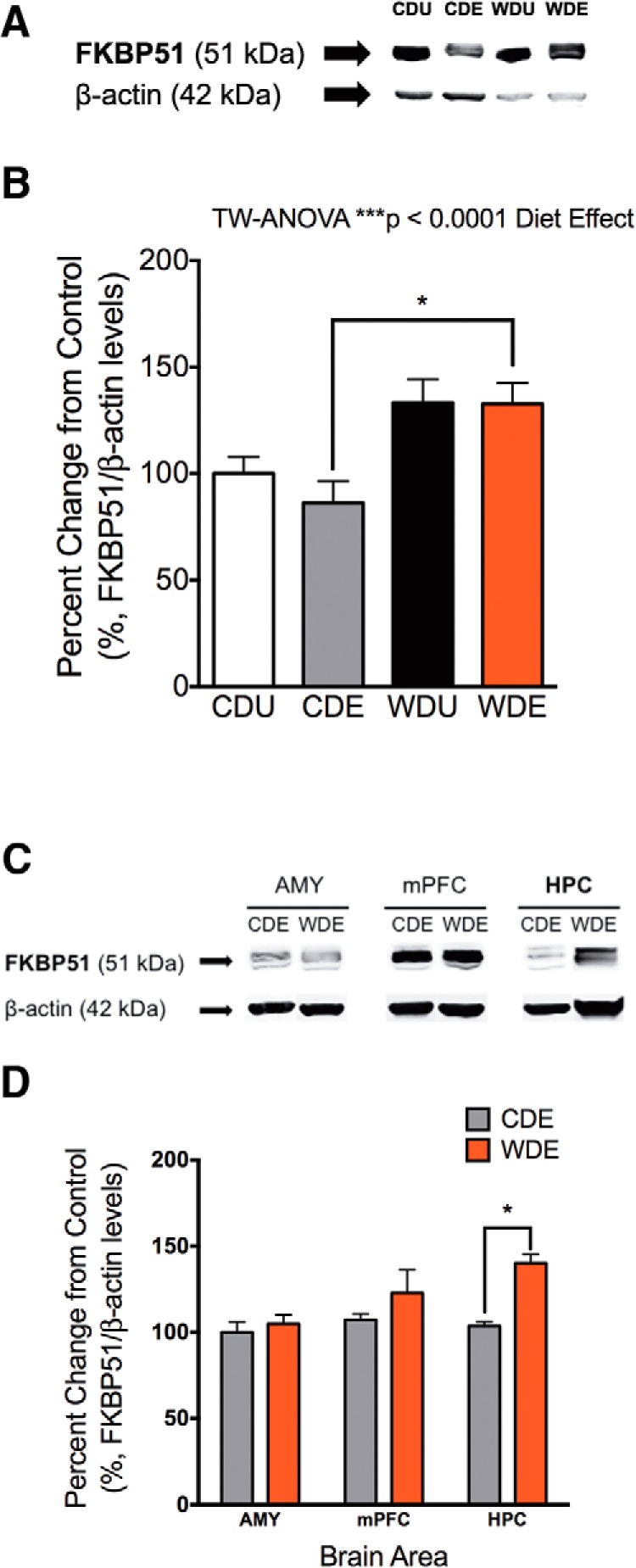
WD increases FKBP51 protein levels in the hippocampus. ***A***, Representative Western blot shows increased FKBP51 levels in the brains of the rats that consumed the WD. ***B***, Analyses showed a significant main effect of the diet on brain FKBP51 levels (diet effect: *F*_(1,19)_ = 15.45, *p* = 0.0009), PS exposure (*F*_(1,19)_ = 0.51, *p* = 0.49), or interaction between diet and PS exposure (*F*_(1,19)_ = 0.42, *p* = 0.52) did not show significant main effects on FKBP51 levels. ***C***, Representative blot from micropunched anxiety-related brain regions showed distinctive FKBP51 levels. ***D***, Analyses revealed a significant increase in FKBP51 levels in the hippocampus of the WDE rats when compared with CDE rats (*F*_(1,16)_ = 9.88; *p* = 0.0063, *p* = 0.021). Diet did not alter FKBP51 levels in the amygdala or medial prefrontal cortex. Although it did not reach statistical significance, we found a trend for more prefrontal cortex FKBP51 levels when compared with the amygdala and the hippocampus (*F*_(2,16)_ = 3.58, *p* = 0.052). No significant interactions between diet and the studied brain regions were revealed (*F*_(2,16)_ = 2.44, *p* = 0.12). Data are expressed as the mean ± SEM. *p < 0.05. Amygdala (AMY), *n* = 3–4 rats; medial prefrontal cortex (mPFC), *n* = 3–4; HPC, *n* = 4–5.

To explore clinically relevant signaling pathways implicated in PTSD, we examined the effects of PS exposure and the WD on the FKBP51 levels in the brain. In agreement with previous studies, we found that the WD increased FKBP51 protein levels in the brain (diet: *F*_(1,19)_ = 15.45, *p* = 0.0009; Fig. 6*A*,*B*). We found that PS exposure or interaction between diet and PS did not show significant effects on brain FKBP51 protein levels (PS: *F*_(1,19)_ = 0.51, *p* = 0.49; interaction: *F*_(1,19)_ = 0.42, *p* = 0.52). To gain insights into putative mechanisms associated with the anxiety-like behaviors following PS exposure, we measured FKBP51 levels in brain regions associated with the stress response, including the amygdala, the medial prefrontral cortex, and the hippocampus (Fig. 6*C*). Analyses revealed a significant increase in FKBP51 levels in the hippocampus of the WDE rats when compared with animals exposed to PS and consuming the CD (diet: *F*_(1,16)_ = 9.88, *p* = 0.0063; *post hoc* test, *p* = 0.0210; Fig. 6*D*). We found no significant main effects of the brain region or interactions between diet and brain regions on FKBP51 protein levels (brain region: *F*_(2,16)_ = 3.58, *p* = 0.052; interaction: *F*_(2,16)_ = 2.44, *p* = 0.12).

### The hippocampus shows distinctive and asymmetric vulnerabilities to the Western high-fat diet and traumatic stress exposure

**Figure 7.**
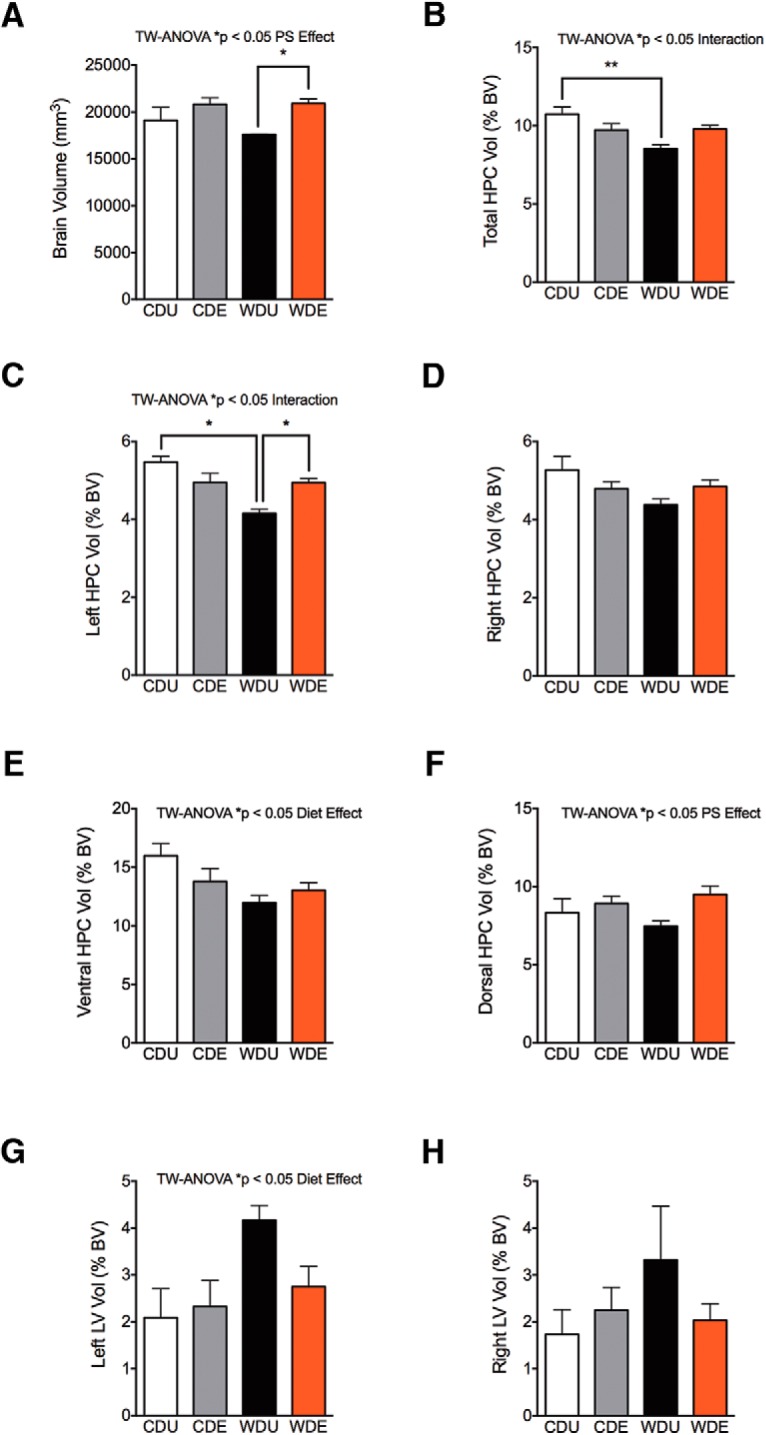
WD and traumatic stress exposure alter hippocampal volume in a region-specific manner. ***A***, We found a significant main effect of PS on total brain volume (*F*_(1,11)_ = 11.31, *p* = 0.0063). *Post hoc* comparisons indicated that PS exposure increased the brain volume in WDE rats when compared with CDU rats (*p* = 0.036). TW-ANOVA revealed no significant main diet effects (*F*_(1,11)_ = 0.91, *p* = 0.36) or interactions (*F*_(1,11)_ = 1.13, *p* = 0.31) on total brain volume. ***B***, WDU rats showed reduced HPC volumes when compared with CDU rats (diet effect: *F*_(1,11)_ = 9.59, *p* = 0.010; *post hoc* test, *p* = 0.0080). Although analyses revealed no significant main PS effects (*F*_(1,11)_ = 0.13, *p* = 0.73), we found a significant interaction between diet and PS exposure (*F*_(1,11)_ = 10.62, *p* = 0.0076) on total brain volume. ***C***, The left HPC volume was significantly affected by the diet (*F*_(1,11)_ = 7.34, *p* = 0.020) and PS exposure (*F*_(1,11)_ = 5.88, *p* = 0.034). *Post hoc* comparisons revealed reduced HPC volumes in WDU rats when compared with CDU rats (WDU vs CDU, *p* = 0.022) and WDE rats (WDU vs WDE, *p* = 0.015). We also found significant interactions between the diet and PS exposure (*F*_(1,11)_ = 7.38, *p* = 0.020). ***D***, The diet type (*F*_(1,11)_ = 3.74, *p* = 0.079) and PS exposure (*F*_(1,11)_ = 0.00091, *p* = 0.98) did not have a significant effect on right HPC volumes (interaction: *F*_(1,11)_ = 4.83, *p* = 0.050). ***E***, We found reduced ventral HPC volumes in the rats that consumed the WD (*F*_(1,11)_ = 6.93; *p* = 0.023). We found no significant main effects of PS exposure (*F*_(1,11)_ = 0.39, *p* = 0.54) or interactions (*F*_(1,11)_ = 3.27, *p* = 0.098) on ventral HPC volumes. ***F***, Interestingly, PS exposure led to increased dorsal HPC volumes (*F*_(1,11)_ = 7.45; *p* = 0.020). We found no significant main effects of the diet type (*F*_(1,11)_ = 0.19, *p* = 0.67) or significant interactions between diet and PS exposure (*F*_(1,11)_ = 1.23, *p* = 0.29). ***G***, The WD had a significant effect on increasing the left lateral ventricle volume (*F*_(1,11)_ = 5.95; *p* = 0.033). We found no significant main effects of PS exposure (*F*_(1,11)_ = 1.33, *p* = 0.27) or interactions (*F*_(1,11)_ = 2.59, *p* = 0.14). ***H***, We found no significant main effects of diet (*F*_(1,11)_ = 1.23, *p* = 0.29), PS exposure (*F*_(1,11)_ = 0.39, *p* = 0.54), or interactions (*F*_(1,11)_ = 2.13, *p* = 0.17) on the right lateral ventricular volume. *p < 0.05, **p < 0.01; *n* = 3–5 brains/group. BV, Brain volume; LV, lateral ventricle.

We performed MRI measurements of brain volumes to further determine the effects of the WD and PS exposure on hippocampal and ventricular structure. We found that PS exposure increased the total brain volume (PS: *F*_(1,11)_ = 11.31, *p* = 0.0063; Fig. 7*A*). *Post hoc* comparisons indicated that PS exposure increased the brain volume in the rats that consumed the WD when compared with unexposed controls (WDE vs WDU: *p* = 0.036). TW-ANOVA revealed no significant main diet effects or interaction effects on total brain volume (diet: *F*_(1,11)_ = 0.91, *p* = 0.36; interaction: *F*_(1,11)_ = 1.13, *p* = 0.31).

In agreement with a previous report (Kalisch et al., 2006), we normalized our volumetric data for brain volume, which inherently corrects for the influences of body weight. For the normalized total hippocampal volume, we found a significant main effect of the diet (*F*_(1,11)_ = 9.59, *p* = 0.010; Fig. 7*B*). *Post hoc* comparisons indicated that WDU rats had reduced hippocampal volumes when compared with CDU rats (*p* = 0.0080). Although analyses revealed no significant main PS effects, we found a significant interaction between diet and PS exposure on the total hippocampus volume (PS: *F*_(1,11)_ = 0.12, *p* = 0.73; interaction: *F*_(1,11)_ = 10.62, *p* = 0.0076). This interaction supports our biometric and behavioral findings demonstrating that the effects of PS exposure differ between CD and WD rats. TW-ANOVA revealed significant main effects of diet, PS exposure, and interactions between these factors on the left HPC volume (diet: *F*_(1,11)_ = 7.34, *p* = 0.020; PS: *F*_(1,11)_ = 5.88, *p* = 0.034; interaction: *F*_(1,11)_ = 7.38, *p* = 0.020; Fig. 7*C*). *Post hoc* comparisons revealed significant differences in left HPC volume between WDE and WDU rats (*p* = 0.015), CDE and WDU rats (*p* = 0.020), and CDU and WDU rats (*p* = 0.022). Conversely, analyses indicated no significant main effects of the diet, PS exposure effects, or significant interactions on right HPC volume (diet: *F*_(1,11)_ = 3.74, *p* = 0.079; PS: *F*_(1,11)_ = 0.00091, *p* = 0.98; interaction: *F*_(1,11)_ = 4.83, *p* = 0.0503; Fig. 7*D*). We found that the diet type had a significant impact on the anteroposterior (AP) hippocampal volume (diet: *F*_(3,90)_ = 5.54; *p* = 0.0016; data not shown). Detailed AP analyses revealed that diet had a significant effect on the ventral hippocampal volume (diet: *F*_(1,11)_ = 6.93; *p* = 0.023; Fig. 7*E*). We found no significant main effects of PS exposure or interactions on the ventral hippocampal volume (PS: *F*_(1,11)_ = 0.39, *p* = 0.54; interaction: *F*_(1,11)_ = 3.27, *p* = 0.098). Notably, PS exposure led to larger dorsal hippocampal volumes in WDE rats when compared with WDU rats (PS: *F*_(1,11)_ = 7.45; *p* = 0.020; Fig. 7*F*). Analyses indicated no significant main effects of the diet or interactions (diet: *F*_(1,11)_ = 0.19, *p* = 0.67; interaction: *F*_(1,11)_ = 1.23, *p* = 0.29) on the dorsal hippocampal volume.

**Figure 8.**
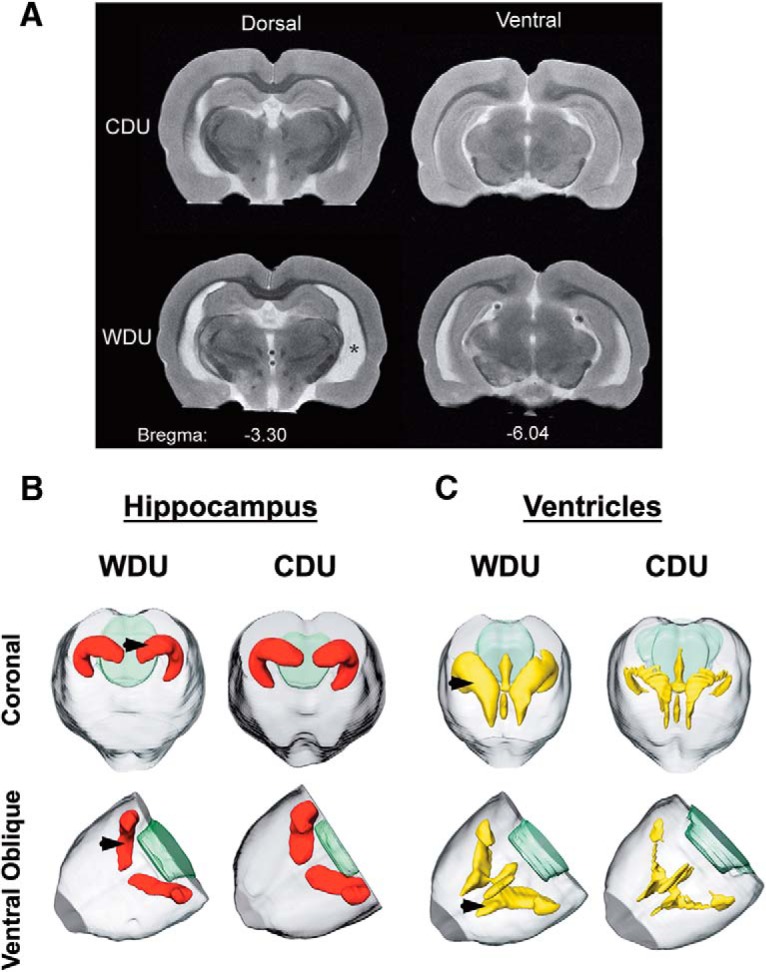
WD consumption led to hippocampal atrophy and ventricular enlargement. ***A***, Representative MR images showing reductions in the anteroposterior hippocampal volume in the rats that consumed the WD. The asterisk indicates larger lateral ventricles in the rats that consumed the WD. ***B***, ***C***, 3D coronal and ventral oblique MRI reconstructions show the impact of the WD on the hippocampal (***B***; red, arrowheads) and ventricular (***C***; yellow, arrowheads) volumes.

We found no significant main effects or interactions on the total ventricular volume (diet: *F*_(1,11)_ = 4.32, *p* = 0.0.062; PS: *F*_(1,11)_ = 0.11, *p* = 0.74; interaction: *F*_(1,11)_ = 2.62, *p* = 0.13; data not shown). We observed similar findings on the effect of diet and PS on the third ventricular volume (diet: *F*_(1,11)_ = 2.27, *p* = 0.16; PS: *F*_(1,11)_ = 0.40, *p* = 0.54; interaction: *F*_(1,11)_ = 0.20, *p* = 0.66; data not shown). Interestingly, the rats that consumed the WD showed enlarged left lateral ventricle volumes (*F*_(1,11)_ = 5.95, *p* = 0.033; Fig. 7*G*). Analyses indicated no significant main effects of PS exposure (PS: *F*_(1,11)_ = 1.33, *p* = 0.27; interaction: *F*_(1,11)_ = 2.59, *p* = 0.14). In contrast, we found no significant main effects of diet, PS exposure, or interactions on the right lateral ventricular volume (diet: *F*_(1,11)_ = 1.23, *p* = 0.29; PS: *F*_(1,11)_ = 0.39, *p* = 0.54; interaction: *F*_(1,11)_ = 2.13, *p* = 0.17; Fig. 7*H*). MRI showed smaller hippocampal formation and larger ventricular volumes in the rats that consumed the WD when compared with the CD (Fig. 8*A*). Three-dimensional (3D) MRI reconstructions confirmed the impact of the WD on the hippocampal and ventricular structures. Coronal and ventral oblique reconstructed planes confirmed the smaller hippocampal volume (Fig. 8*B*) and larger ventricular volume (Fig. 8*C*) in a representative WDU rat brain.

### Consumption of a WD reduces hippocampal vascular integrity

**Figure 9.**
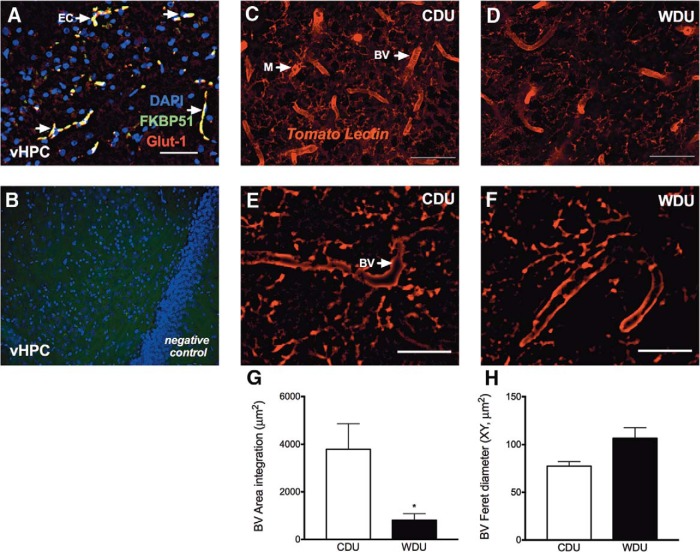
A WD alters neurovascular integrity in the hippocampus. ***A***, Representative photomicrograph showing FKBP51 expression in hippocampal Glut-1^+^ endothelial cells in a rat that consumed the WD. ***B***, Immunoreactivity specificity was confirmed by omitting primary antibodies. ***C***, ***D***, Fluorescence photomicrographs show that the hippocampus of rats that consumed the CD (***C***) had more tomato-lectin^+^ (TL) staining compared with the hippocampus of animals consuming the WD (***D***). ***E***, ***F***, Representative photomicrographs depict smaller blood vessel diameter in the rats that consumed the CD (***E***) when compared with rats that consumed the WD (***F***). ***G***, Analyses revealed reduced TL^+^ staining area in the animals consuming the WD (*t*_(6)_ = 2.69, *p* = 0.036). ***H***, No significant differences were observed in the blood vessel diameter when diet groups were compared. BV, Blood vessel; M, microglia. *n* = 4 brains per group. Student’s *t* test, *p < 0.05. Scale bars, 25 μm.

We found hippocampal FKBP51 immunoreactivity in cells closely resembling the outline of glial projections and microvessels (data not shown). In order to confirm that this distinct FKBP51 immunoreactivity was in fact associated with hippocampal microvessels, we performed double-labeling immunohistochemistry. We found FKBP51 expression in Glut-1^+^ endothelial cells, confirming that FKBP51 was proximal to microvessels (Fig. 9*A*). Primary antibody omission controls confirmed the specificity of the immunohistochemical labeling (Fig. 9*B*). These observations led us to determine the levels of tomato lectin (TL) staining to investigate the effects of the diet on hippocampal microvasculature integrity. TL is a protein with specific affinity for sugar residues found in endothelial and microglial cells. We found increased TL labeling within the hippocampus of unexposed rats receiving the CD (Fig. 9*C*) compared with unexposed rats consuming the WD (Fig. 9*D*). Representative micrographs showed smaller blood vessel diameter in the rats that consumed the CD (Fig. 9*E*) when compared with the rats that received the WD (Fig. 9*F*). Histological analyses revealed a significant reduction in TL-positive blood vessels in rats consuming the WD (*t*_(6)_ = 2.69, *p* = 0.036; Fig. 9*G*). No significant differences were observed in the blood vessel feret diameter between diet groups (*p* > 0.05; Fig. 9*H*). We found no significant differences in peripheral (white blood cell counts) and central (hippocampal microglia cell counts) inflammation biomarkers when comparing diet groups (data not shown; for both, *p* > 0.05).

### Obesity biomarkers are associated with brain and behavioral signatures of stress susceptibility

**Figure 10.**
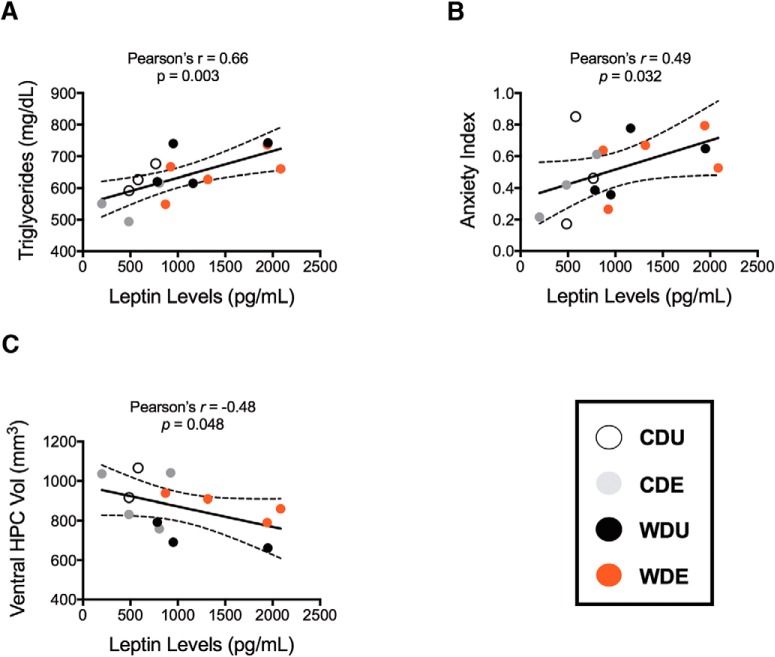
Pearson’s correlations show significant associations between obesity and stress biomarkers. ***A–C***, Scatter plots show a significant relationship between plasma triglyceride and leptin levels (***A***), leptin levels and the EPM anxiety index (***B***), and leptin levels and ventral hippocampal volume (***C***). Pearson’s correlation was significant at *p* < 0.05. Solid line, Linear regression; outer dashed line, 95% confidence intervals; CDU, clear circles; CDE, gray circles; WDU, black circles; WDE rats, red circles.

The plasma triglyceride levels showed a robust significant correlation with leptin levels in plasma (*r* = 0.66, *p* = 0.003; Fig. 10*A*). A similar and significant relationship was found between leptin levels and body weight (*r* = 0.56, *p* = 0.023; data not shown). We found a significant correlation between leptin levels and the EPM anxiety index (*r* = 0.49, *p* = 0.032; Fig. 10*B*). Notably, analyses revealed that leptin levels were negatively associated with the ventral hippocampal volume (*r* = −0.48, *p* = 0.048; Fig. 10*C*).

## Discussion

In this study, we sought to determine how consumption of a Western high-fat diet during adolescence impacts post-traumatic stress responsiveness. We demonstrate that the consumption of a WD contributes to neurobehavioral and neuroanatomical alterations following short-term exposure to PS. In this report, we present further experimental evidence suggesting that these maladaptive stress responses may be associated with the altered metabolism and increased FKBP51 levels in the hippocampus. Notably, this study provides new compelling evidence of the lateralization of diet and stress effects in the HPC and lateral ventricles. We present translationally relevant neural substrates that may predispose the obese brain to anxiety-related disorders following traumatic stress. Finally, the findings reported here provide new arguments in favor of the hypothesis proposing that gene–environment interactions during adolescence play a critical role in the neurobiological development of lifelong stress responses. Table 4 summarizes the main findings of the study.

**Table 4:** Summary of findings

	WD effect	PS effect	WD × PS interactions
Biometrics			
Body weight	↑	↑	n.s.
Caloric consumption	↑	↑	n.s.
Fasting blood glucose	n.s.	n.s.	n.s.
Postprandial blood glucose	n.s.	n.s.	s.
Behavioral			
ASR	n.s.	↑	n.s.
ASR habituation	↑	n.s.	n.s.
Prepulse inhibition	n.s.	n.s.	n.s.
OF center entries and duration	n.s.	↑	n.s.
OF anxiety index	n.s.	↑	n.s.
OF distance traveled	n.s.	↓	n.s.
EPM open arm entries and duration	↓	↓	n.s.
EPM anxiety index	↑	↑	n.s.
EPM head dipping zone	↓	n.s.	n.s.
EPM stretched postures	n.s.	↑	n.s.
FST mobility	n.s.	n.s.	n.s.
Endocrine			
Corticosterone levels	n.s.	n.s.	n.s.
FKBP51 protein levels	↑	n.s.	n.s.
Plasma leptin levels	↑	n.s.	n.s.
Plasma triglycerides	n.s.	n.s.	n.s.
Anatomical			
Brain volume	n.s.	↑	n.s.
HPCV	↓	n.s.	s.
Ventral HPCV	↓	n.s.	n.s.
Dorsal HPCV	n.s.	↑	n.s.
Left HPCV	↓	n.s.	*s*.
Right HPCV	n.s.	n.s.	n.s.
Left lateral ventricle volume	↑	n.s.	n.s.
Right lateral ventricle volume	n.s.	n.s.	n.s.
Histological			
HPC microglia density/area	n.s.	n.d.	n.d.
HPC blood vessel density/area	↓	n.d.	n.d.

HPCV, HPC volume; OF, open field; s., significant main effect; n.s., nonsignificant main effect; n.d., not determined. Our cross-scale study design allowed for the identification of fundamental relationships among neuroanatomical and neurovascular structure, neuroendocrine function, and behavior. Table shows statistically significant main effects of the WD, PS, or interactions as determined by two-way ANOVA. Arrows depict whether the WD or PS significantly altered the outcome measures and the direction of change.

Despite the growing epidemiological evidence showing associations between obesity and PTSD (Scott et al., 2008; Perkonigg et al., 2009; Pagoto et al., 2012; Johannessen and Berntsen, 2013; Maguen et al., 2013; Mitchell et al., 2013; Kubzansky et al., 2014; Bartoli et al., 2015; Smith et al., 2015), the neural basis of this relationship remains largely understudied. In this study, by subjecting adolescent rats to a typical Western-type obesogenic diet, we were able to reproduce various metabolic phenotypes associated with obesity. For instance, we observed postprandial hypoglycemia in the animals fed the WD, a condition commonly associated with anxiety and emotionality in both humans (Strachan et al., 2000) and rats (McNay, 2015). Not surprisingly, we report increased leptin levels in the rats that consumed the WD, and a robust correlation between TG and leptin. This finding is in agreement with those of previous studies reporting TG effects on leptin resistance (Chen et al., 2002; Banks et al., 2004). Although the regulatory actions of leptin on energetics and food intake have been extensively reported, emerging data support its importance in neural development and plasticity (Dhar et al., 2014), hippocampal function, and psychiatric disorders (Sharma et al., 2010; Guo et al., 2013; Tyree et al., 2016), including PTSD (Liao et al., 2004; Farr et al., 2015).

In addition to experiencing alterations in cognition and arousal, youth with PTSD experience anxiety and distressing traumatic memories. A major finding of our study is that the rats that consumed the WD exhibited increased susceptibility to anxiety-like behaviors following traumatic stress. This study used a habituation-test comprehensive behavioral profiling approach to determine the emotional reactivity to traumatic stress. A growing body of evidence shows that repeated behavioral testing induces experience-dependent behavioral alterations, which may represent indices for contextual memory acquisition, emotionality, and fear (File, 1993; Treit et al., 1993; Fernandes and File, 1996; Bertoglio and Carobrez, 2000; Bertoglio et al., 2006; Stern et al., 2010; Gianlorenço et al., 2012; Chegini et al., 2014). Importantly, it has been reported that novelty-seeking behaviors may predict vulnerability to anxiety-like behaviors following stress, particularly in inbred rats such as the Lewis strain used in this study (Vollmayr et al., 2004; Shumake et al., 2005; Pawlak et al., 2008; Goswami et al., 2012). Thus, in agreement with these reports, we reasoned that a habituation-test paradigm would reduce confounding factors associated with novelty (Pawlak et al., 2008; Schneider et al., 2011). Further, re-exposure to behavioral testing was intended to measure key aspects of emotional memories associated with PTSD (Berardi et al., 2014). In particular, we intended to investigate those behaviors associated with aversion, anxiety, fear, and context generalizations.

Intriguingly, we found that the animals that consumed the WD exhibited anxiety-like behaviors in the OFT only following traumatic stress exposure. It is possible that the increased post-traumatic stress reactions in the OFT are related to enhanced emotional memory and fear generalization since this environment resembles the one in which the odor threat was originally encountered. By using indices of anxiety in the elevated plus maze, we validated the anxiogenic effects of the combined exposure to the WD and traumatic stress. Interestingly, the effects of high-fat diets on anxiety-like behaviors remains highly controversial, with studies showing that the consumption of high-fat diets can induce anxiolytic effects (McNeilly et al., 2015; Maniam et al., 2016), with others showing anxiogenic effects (Sharma et al., 2012; Sivanathan et al., 2015). This discrepancy can be partly explained by the duration of high-fat diet feeding, and by the composition and source of the dietary fats. Further, the genetic background and developmental stage of the animal and the behavioral paradigms used are major contributing factors to the development of anxiety-like behaviors in rats. In this study, we used typical Western diet oil sources and fatty acid compositions in a Lewis rat model of PTSD. Interestingly, this rat strain exhibits blunted neuroendocrine stress responses that are similar to those reported in children and adolescents with comorbid cognitive impairments and traumatic stress history (Nugent et al., 2007; Yehuda et al., 2007; Danielson et al., 2015). A thought-provoking interpretation of our data is that the WD affected the developmental trajectories of the neural circuitry underlying emotional learning. The results of our habituation-test paradigm suggest that the consumption of a WD may facilitate context conditioning and retrieval while impairing the extinction of emotional memories. This idea is supported by an elegantly designed study showing exaggerated aversion and fear memories in adolescent rats exposed to a high-fat diet (Boitard et al., 2015).

In agreement with the individual variations of symptoms observed in PTSD patients, we found distinctive phenotypes following exposure to a traumatic predator odor threat. The lack of increased ASR exhibited by animals consuming the WD may seem to contradict the relevance of our findings for post-traumatic stress responses, since startle elevation is one of the most commonly cited features of PTSD (Morgan et al., 1996; Cohen et al., 2006a; Rasmussen et al., 2008). However, few studies have reported blunted or even reduced ASR in patients experiencing PTSD (Ornitz and Pynoos, 1989; Medina et al., 2001; Grillon and Baas, 2003) and in animal models of stress (Conti and Printz, 2003; Adamec et al., 2006; Beck et al., 2008). It is also likely that the dampened startle reactivity in the rats that consumed the WD is a result of competing interactions between the diet and traumatic stress, since high-fat diets alone can impair sensorimotor gating (Labouesse et al., 2013; Wakabayashi et al., 2015). Further, the blunted startle reactivity may imply alterations in sensory thresholds for eliciting ASR, motor deficits, and peripheral mechanisms. One such mechanism is inflammation, which has been shown to reduce startle responses through interleukin-1 production in the immune-sensitive Lewis rats (Beck and Servatius, 2006). Thus, it is reasonable that in priming the immune system (Erion et al., 2014), the WD reduces the responsivity of the ASR. In contrast with other studies showing the effect of high-fat diet consumption on the FST (Krishna et al., 2015; Pase et al., 2015), we found no significant effects on FST behaviors during adulthood. Although this lack of effect may be confounded by differences in body fat composition and buoyancy, this finding is not altogether surprising, as the FST has been reported to measure ways of coping with an inescapable stressor rather than behaviors associated with despair and depression (Veenema et al., 2003). Notably, when comparing the effect of the diet in time, we found that rats that consumed the WD exhibited increased immobility during adulthood. This effect confirms that age may play a role in stress coping strategies and that perhaps prolonged exposure to the WD is required to produce depressive-like behaviors in this rat strain (Meyza et al., 2011). It is also possible that the WD facilitates retrieval of emotional memories during re-exposure to the FST. Together, our behavioral data show that traumatic experiences may not elicit uniform effects on stress-related behaviors in animals consuming an obesogenic diet.

One intriguing question arising from the behavioral data is how the consumption of a Western high-fat diet may elicit maladaptive traumatic stress responses. The *FKBP5* gene encodes for the chaperone protein FKBP51 and regulates the sensitivity of the stress response (Hoeijmakers et al., 2014; Schmidt et al., 2015). Specifically, FKBP51 attenuates glucocorticoid receptor-dependent signaling and in turn shuts down the HPA axis activation. FKBP51 is highly sensitive to environmental stressors and has been implicated in several psychiatric conditions, particularly PTSD (Binder et al., 2008; Young et al., 2015; Yehuda et al., 2016). In agreement with these findings, our data point to FKBP51 as an important player in post-traumatic stress responses. As shown in previous studies, we found that FKBP51 levels are sensitive to high-fat diets and obesogenic phenotypes (Yang et al., 2012; Castro et al., 2013; Balsevich et al., 2014, 2016). Importantly, we report a highly selective increase in FKBP51 levels in the hippocampus of the rats that consumed the WD. We provide further evidence supporting a potential role for this protein in vascular integrity and angiogenesis (Srivastava et al., 2015). Thus, the regulation of endothelial glucocorticoid signaling via FKBP51 may be a potential biologic mechanism underlying the vascular deficits observed in rats that consumed the WD. Our histological findings showing a reduced number of blood vessels in the rats that consumed the WD are supported by studies that demonstrate that consumption of a high-saturated fat diet induces marked alterations in the brain vascular integrity (Freeman and Granholm, 2012). Similar effects are observed in the hippocampus of a commonly used diabetic rat model (Beauquis et al., 2010). Our results confirm a potential role for high-fat diets and FKBP51 in determining hippocampal maturation and risk for post-traumatic stress responses. This idea is supported by studies in humans demonstrating a strong inverse relationship between FKBP51 transcript levels and dendritic spine density (Young et al., 2015) and stress circuitry connectivity (Holz et al., 2015). Together, the findings reported in the present study expand on these observations and propose a new mechanism for the impairments in hippocampal function observed in individuals who consume high-fat diets.

To our knowledge, this is the first animal study to demonstrate associations between WD consumption during adolescence and reduced HPC volume. Our structural neuroimaging results are consistent with studies demonstrating that the HPC is highly sensitive to the effects of environmental stressors (Sapolsky, 1986; Mondelli et al., 2011; Booij et al., 2015). The HPC exerts strong regulatory control of the stress response (Sapolsky et al., 1984; Jacobson and Sapolsky, 1991; Dedovic et al., 2009). Here, we present new evidence showing the combined effects of traumatic stress and a WD on the HPC volume. Several lines of evidence demonstrate that the hippocampus volume is reduced in PTSD (Karl et al., 2006; Woon and Hedges, 2008; Woon et al., 2010; Golub et al., 2011; Kühn and Gallinat, 2013). Strikingly, as shown in a recent study in humans (Jacka et al., 2015), we present new evidence that the consumption of a WD selectively alters the volume of the left HPC in rats. We show that traumatic stress has a differential impact on the left HPC volume of the rats that consumed the WD. A trial-limited optogenetics study in mice supports our finding by showing that only the left HPC is required for associative spatial long-term memory (Shipton et al., 2014), an important outcome evaluated in our habituation-test behavioral paradigm and implicated in the pathophysiology of PTSD. The left HPC has been also implicated in memory reconsolidation by detecting mismatches between actual and learned events (Forcato et al., 2016). We report reduced ventral HPC volumes in the rats that consumed the WD. Lesion and optogenetics studies have demonstrated an essential role for the ventral HPC in anxiety and fear (Kjelstrup et al., 2002; Kheirbek et al., 2013). Further, a series of well designed studies in humans demonstrated the importance of the ventral HPC in anxiety-related behaviors associated with approach–avoidance conflicts similar to the ones tested in this study (Bach et al., 2014). Although there remains a debate as to whether smaller hippocampal volume represents a premorbid or an acquired trait in PTSD, our results suggests that a structurally reduced hippocampus may predispose rats to exhibit aberrant post-traumatic stress responses. Notably, we report that the rats that consumed the WD showed significant ventricular enlargements. This ventricular enlargement was particularly evident in the left lateral ventricle, supporting a selective vulnerability to the effects of the WD on the left side of the brain. It is important to note that ventriculomegaly is a commonly observed pathologic biomarker of neurodegenerative disorders and aging, and results from passive enlargement of the ventricles following shrinkage of brain parenchyma (Apostolova et al., 2012). Together, our imaging results support a growing body of evidence demonstrating that the left HPC exhibits a selective vulnerability to early-life stress and metabolic alterations (Gorka et al., 2014; Wang et al., 2015), which may serve as a promising early diagnosis or intervention biomarker. In the context of PTSD, we hypothesize that the impact of a WD on the left and ventral hippocampal formation is such that it would affect memory retrieval and reconsolidation, resulting in anxiety and fear-related behaviors. Further, these diet-induced structural changes may hinder the ability of the HPC to shut off the HPA axis, resulting in exaggerated stress reactions. In turn, the inability of the HPC in shutting off the stress response will lead to additional hippocampal structural impairments (i.e., decreased dendritic branching), thereby inducing a vicious cycle of HPC degeneration and atrophy. Together, we hypothesize that elevated FKBP51 levels in hippocampal microglia and endothelial cells reduce the sensitivity to glucocorticoid signaling and lipid metabolism deregulation, resulting in sustained inflammation and altered neurovascular integrity. This is expected to result in structural changes within the limbic stress neurocircuitry (via cell death, axonal/myelin degeneration, and reduced neurogenesis mechanisms). This faulty stress circuitry may predispose the brain to aberrant stress responsiveness and impose a greater vulnerability to PTSD (van Rooij et al., 2015).

While our experimental approach did not provide definitive mechanistic information, it clarifies which aspects of diet-induced obesity may be associated with post-traumatic stress responses and should contribute to generate detailed hypotheses for mechanistic investigation. Ongoing studies in our laboratory are focused to determine whether FKBP51 levels predispose the brain to traumatic stress by virtue of regulating the blood–brain barrier permeability, inflammation, neuronal turnover, and, ultimately, hippocampal structure. Further experiments explicitly testing the hypothesis of a negative relationship between hippocampal volume and anxiety, and the impact of underlying environmental factors are also warranted. Future studies will also benefit from using alternative neuroimaging modalities and longitudinal studies to investigate the long-term impact of nutrition on the neurocircuitry of stress. Although the experimental PTSD model and behavioral paradigms used in this study are well established and validated, there are limitations in extrapolating these findings from experimental animals to humans. Animal models are restricted to the evaluation of “isolated” behavioral manifestations and cannot reflect the full PTSD construct. The proper interpretation of animal models of PTSD thus remains a challenge and warrants further investigation. Nevertheless, this study presents some of the first experimental efforts to incorporate behavioral responses associated with contextual memory alterations following traumatic stress (Ritov and Richter-Levin, 2014), a core manifestation of PTSD.

There has been little research conducted that addresses the mental health care needs of our growing overweight and obese population. Despite clinical evidence supporting a strong link between exposure to environmental stressors and the ontogeny of psychiatric disorders in children and adolescents, diet remains a largely unexplored vulnerability factor for stress-related psychiatric disorders. Our study shows that typical Western dietary patterns may have long-term negative consequences on how the brain copes with stress later in life. Further, this study provides a conceptual framework focused on understanding the neurobiological basis of stress-related health disparities. Understanding how unhealthy nutritional practices during adolescence impair brain maturation and behavior can inform novel therapeutic strategies and lifestyle changes to improve the future of mental health.

## Synthesis

The decision was a result of the Reviewing Editor Jeffrey Blaustein and the peer reviewers coming together and discussing their recommendations until a consensus was reached. A fact-based synthesis statement explaining their decision and outlining what is needed to prepare a revision is listed below. The following reviewers agreed to reveal their identity: Carsten Wotjak, Kellie Tamashiro

## References

[B1] Abel EL (1994) A further analysis of physiological changes in rats in the forced swim test. Physiol Behav 56:795–800. 7800751 10.1016/0031-9384(94)90245-3

[B2] Adamec R, Strasser K, Blundell J, Burton P, McKay DW (2006) Protein synthesis and the mechanisms of lasting change in anxiety induced by severe stress. Behav Brain Res 167:270–286. 10.1016/j.bbr.2005.09.019 16256211

[B3] Alemany S, Moya J, Ibáñez MI, Villa H, Mezquita L, Ortet G, Gastó C, Fañanás L, Arias B (2016) Research Letter: childhood trauma and the rs1360780 SNP of FKBP5 gene in psychosis: a replication in two general population samples. Psychol Med 46:221–223. 10.1017/S0033291715001695 26399750

[B130] Apostolova LG, Green AE, Babakchanian S, Hwang KS, Chou Y-Y, Toga AW, Thompson PM (2012) Hippocampal atrophy and ventricular enlargement in normal aging, mild cognitive impairment (MCI), and Alzheimer Disease. Alzheimer Dis Assoc Disord 26:17–27. 22343374 10.1097/WAD.0b013e3182163b62 PMC3286134

[B4] Bach DR, Guitart-Masip M, Packard PA, Miró J, Falip M, Fuentemilla L, Dolan RJ (2014) Human hippocampus arbitrates approach-avoidance conflict. Curr Biol 24:541–547. 10.1016/j.cub.2014.01.046 24560572 PMC3969259

[B5] Balsevich G, Uribe A, Wagner KV, Hartmann J, Santarelli S, Labermaier C, Schmidt MV (2014) Interplay between diet-induced obesity and chronic stress in mice: potential role of FKBP51. J Endocrinol 222:15–26. 10.1530/JOE-14-0129 24781256

[B6] Balsevich G, Baumann V, Uribe A, Chen A, Schmidt MV (2016) Prenatal exposure to maternal obesity alters anxiety and stress coping behaviors in aged mice. Neuroendocrinology 103:354–368. 10.1159/000439087 26279463

[B7] Banks WA, Coon AB, Robinson SM, Moinuddin A, Shultz JM, Nakaoke R, Morley JE (2004) Triglycerides induce leptin resistance at the blood-brain barrier. Diabetes 53:1253–1260. 15111494 10.2337/diabetes.53.5.1253

[B8] Bartoli F, Crocamo C, Alamia A, Amidani F, Paggi E, Pini E, Clerici M, Carrà G (2015) Posttraumatic stress disorder and risk of obesity: systematic review and meta-analysis. J Clin Psychiatry 76:e1253–e1261. 10.4088/JCP.14r09199 26528647

[B9] Beauquis J, Homo-Delarche F, Giroix M-H, Ehses J, Coulaud J, Roig P, Portha B, De Nicola AF, Saravia F (2010) Hippocampal neurovascular and hypothalamic-pituitary-adrenal axis alterations in spontaneously type 2 diabetic GK rats. Exp Neurol 222:125–134. 10.1016/j.expneurol.2009.12.022 20045412

[B10] Beck KD, Servatius RJ (2006) Interleukin-1beta as a mechanism for stress-induced startle suppression in females. Ann N Y Acad Sci 1071:534–537. 10.1196/annals.1364.058 16891613

[B11] Beck KD, Jiao X, Cominski TP, Servatius RJ (2008) Estrus cycle stage modifies the presentation of stress-induced startle suppression in female Sprague-Dawley rats. Physiol Behav 93:1019–1023. 10.1016/j.physbeh.2008.01.012 18281068

[B12] Berardi A, Trezza V, Palmery M, Trabace L, Cuomo V, Campolongo P (2014) An updated animal model capturing both the cognitive and emotional features of post-traumatic stress disorder (PTSD). Front Behav Neurosci 8:142. 10.3389/fnbeh.2014.00142 24808840 PMC4010768

[B13] Bertoglio LJ, Carobrez AP (2000) Previous maze experience required to increase open arms avoidance in rats submitted to the elevated plus-maze model of anxiety. Behav Brain Res 108:197–203. 10701663 10.1016/s0166-4328(99)00148-5

[B14] Bertoglio LJ, Joca SRL, Guimarães FS (2006) Further evidence that anxiety and memory are regionally dissociated within the hippocampus. Behav Brain Res 175:183–188. 10.1016/j.bbr.2006.08.021 16996146

[B15] Binder EB (2009) The role of FKBP5, a co-chaperone of the glucocorticoid receptor in the pathogenesis and therapy of affective and anxiety disorders. Psychoneuroendocrinology 34: Suppl 1:S186–S195. 10.1016/j.psyneuen.2009.05.021 19560279

[B16] Binder EB, Salyakina D, Lichtner P, Wochnik GM, Ising M, Pütz B, Papiol S, Seaman S, Lucae S, Kohli MA, Nickel T, Künzel HE, Fuchs B, Majer M, Pfennig A, Kern N, Brunner J, Modell S, Baghai T, Deiml T, et al. (2004) Polymorphisms in FKBP5 are associated with increased recurrence of depressive episodes and rapid response to antidepressant treatment. Nat Genet 36:1319–1325. 10.1038/ng1479 15565110

[B17] Binder EB, Bradley RG, Liu W, Epstein MP, Deveau TC, Mercer KB, Tang Y, Gillespie CF, Heim CM, Nemeroff CB, Schwartz AC, Cubells JF, Ressler KJ (2008) Association of FKBP5 polymorphisms and childhood abuse with risk of posttraumatic stress disorder symptoms in adults. JAMA 299:1291–1305. 10.1001/jama.299.11.1291 18349090 PMC2441757

[B18] Binder EB, Owens MJ, Liu W, Deveau TC, Rush AJ, Trivedi MH, Fava M, Bradley B, Ressler KJ, Nemeroff CB (2010) Association of polymorphisms in genes regulating the corticotropin-releasing factor system with antidepressant treatment response. Arch Gen Psychiatry 67:369–379. 10.1001/archgenpsychiatry.2010.18 20368512

[B19] Boitard C, Maroun M, Tantot F, Cavaroc A, Sauvant J, Marchand A, Layé S, Capuron L, Darnaudery M, Castanon N, Coutureau E, Vouimba R-M, Ferreira G (2015) Juvenile obesity enhances emotional memory and amygdala plasticity through glucocorticoids. J Neurosci 35:4092–4103. 10.1523/JNEUROSCI.3122-14.2015 25740536 PMC6605580

[B20] Booij L, Szyf M, Carballedo A, Frey E-M, Morris D, Dymov S, Vaisheva F, Ly V, Fahey C, Meaney J, Gill M, Frodl T (2015) DNA methylation of the serotonin transporter gene in peripheral cells and stress-related changes in hippocampal volume: a study in depressed patients and healthy controls. PLoS One 10:e0119061 10.1371/journal.pone.0119061 25781010 PMC4363605

[B21] Carobrez AP, Bertoglio LJ (2005) Ethological and temporal analyses of anxiety-like behavior: the elevated plus-maze model 20 years on. Neurosci Biobehav Rev 29:1193–1205. 10.1016/j.neubiorev.2005.04.017 16084592

[B22] Castro G, C Areias MF, Weissmann L, Quaresma PGF, Katashima CK, Saad MJA, Prada PO (2013) Diet-induced obesity induces endoplasmic reticulum stress and insulin resistance in the amygdala of rats. FEBS Open Bio 3:443–449. 10.1016/j.fob.2013.09.002 PMC3829990 24251109

[B23] Chegini H-R, Nasehi M, Zarrindast M-R (2014) Differential role of the basolateral amygdala 5-HT3 and 5-HT4 serotonin receptors upon ACPA-induced anxiolytic-like behaviors and emotional memory deficit in mice. Behav Brain Res 261:114–126. 10.1016/j.bbr.2013.12.007 24333573

[B24] Chen HC, Ladha Z, Farese RV (2002) Deficiency of acyl coenzyme a:diacylglycerol acyltransferase 1 increases leptin sensitivity in murine obesity models. Endocrinology 143:2893–2898. 10.1210/endo.143.8.8941 12130553

[B25] Clay R, Hebert M, Gill G, Stapleton LA, Pridham A, Coady M, Bishop J, Adamec RE, Blundell JJ (2011) Glucocorticoids are required for extinction of predator stress-induced hyperarousal. Neurobiol Learn Mem 96:367–377. 10.1016/j.nlm.2011.06.012 21736945

[B26] Cohen H, Zohar J (2004) An animal model of posttraumatic stress disorder: the use of cut-off behavioral criteria. Ann N Y Acad Sci 1032:167–178. 10.1196/annals.1314.014 15677404

[B27] Cohen H, Zohar J, Gidron Y, Matar MA, Belkind D, Loewenthal U, Kozloversusky N, Kaplan Z (2006a) Blunted HPA axis response to stress influences susceptibility to posttraumatic stress response in rats. Biol Psychiatry 59:1208–1218. 10.1016/j.biopsych.2005.12.003 16458266

[B28] Cohen H, Matar MA, Richter-Levin G, Zohar J (2006b) The contribution of an animal model toward uncovering biological risk factors for PTSD. Ann N Y Acad Sci 1071:335–350. 10.1196/annals.1364.026 16891582

[B29] Cohen H, Kozloversusky N, Alona C, Matar MA, Joseph Z (2012) Animal model for PTSD: from clinical concept to translational research. Neuropharmacology 62:715–724. 10.1016/j.neuropharm.2011.04.023 21565209

[B30] Conti LH, Printz MP (2003) Rat strain-dependent effects of repeated stress on the acoustic startle response. Behav Brain Res 144:11–18. 12946590 10.1016/s0166-4328(03)00061-5

[B131] Contreras CM, Rodríguez-Landa JF, García-Ríos RI, Cueto-Escobedo J, Guillen-Ruiz G, Bernal-Morales B (2014) Myristic acid produces anxiolytic-like effects in Wistar rats in the elevated plus maze. BioMed Research International 2014:492141–492148. 25328885 10.1155/2014/492141 PMC4189847

[B31] Córdova-Palomera A, de Reus MA, Fatjó-Vilas M, Falcón C, Bargalló N, van den Heuvel MP, Fañanás L (2016) FKBP5 modulates the hippocampal connectivity deficits in depression: a study in twins. Brain Imaging Behav. Advance online publication. Retrieved October 31, 2016 10.1007/s11682-015-9503-4. 26801675

[B32] Danielson CK, Hankin BL, Badanes LS (2015) Youth offspring of mothers with posttraumatic stress disorder have altered stress reactivity in response to a laboratory stressor. Psychoneuroendocrinology 53:170–178. 10.1016/j.psyneuen.2015.01.001 25622009 PMC4333024

[B33] Dedovic K, Duchesne A, Andrews J, Engert V, Pruessner JC (2009) The brain and the stress axis: the neural correlates of cortisol regulation in response to stress. Neuroimage 47:864–871. 10.1016/j.neuroimage.2009.05.074 19500680

[B34] Dhar M, Wayman GA, Zhu M, Lambert TJ, Davare MA, Appleyard SM (2014) Leptin-induced spine formation requires TrpC channels and the CaM kinase cascade in the hippocampus. J Neurosci 34:10022–10033. 10.1523/JNEUROSCI.2868-13.2014 25057204 PMC4107395

[B35] Ellsworth KA, Moon I, Eckloff BW, Fridley BL, Jenkins GD, Batzler A, Biernacka JM, Abo R, Brisbin A, Ji Y, Hebbring S, Wieben ED, Mrazek DA, Weinshilboum RM, Wang L (2013) FKBP5 genetic variation: association with selective serotonin reuptake inhibitor treatment outcomes in major depressive disorder. Pharmacogenet Genomics 23:156–166. 10.1097/FPC.0b013e32835dc133 23324805 PMC3784025

[B36] Erion JR, Wosiski-Kuhn M, Dey A, Hao S, Davis CL, Pollock NK, Stranahan AM (2014) Obesity elicits interleukin 1-mediated deficits in hippocampal synaptic plasticity. J Neurosci 34:2618–2631. 10.1523/JNEUROSCI.4200-13.2014 24523551 PMC3921429

[B37] Fani N, Gutman D, Tone EB, Almli L, Mercer KB, Davis J, Glover E, Jovanovic T, Bradley B, Dinov ID, Zamanyan A, Toga AW, Binder EB, Ressler KJ (2013) FKBP5 and attention bias for threat: associations with hippocampal function and shape. JAMA Psychiatry 70:392–400. 10.1001/2013.jamapsychiatry.210 23407841 PMC3732315

[B38] Farr OM, Ko B-J, Joung KE, Zaichenko L, Usher N, Tsoukas M, Thakkar B, Davis CR, Crowell JA, Mantzoros CS (2015) Posttraumatic stress disorder, alone or additively with early life adversity, is associated with obesity and cardiometabolic risk. Nutr Metab Cardiovasc Dis 25:479–488. 10.1016/j.numecd.2015.01.007 25770759 PMC4404181

[B39] Fernandes C, File SE (1996) The influence of open arm ledges and maze experience in the elevated plus-maze. Pharmacol Biochem Behav 54:31–40. 8728536 10.1016/0091-3057(95)02171-x

[B40] File SE (1993) The interplay of learning and anxiety in the elevated plus-maze. Behav Brain Res 58:199–202. 8136046 10.1016/0166-4328(93)90103-w

[B41] Finsterwald C, Steinmetz AB, Travaglia A, Alberini CM (2015) From memory impairment to posttraumatic stress disorder-like phenotypes: the critical role of an unpredictable second traumatic experience. J Neurosci 35:15903–15915. 10.1523/JNEUROSCI.0771-15.2015 26631471 PMC4666915

[B42] Forcato C, Bavassi L, De Pino G, Fernández RS, Villarreal MF, Pedreira ME (2016) Differential left hippocampal activation during retrieval with different types of reminders: an fMRI study of the reconsolidation process. PLoS One 11:e0151381 10.1371/journal.pone.0151381 26991776 PMC4798722

[B43] Francis H, Stevenson R (2013) The longer-term impacts of Western diet on human cognition and the brain. Appetite 63:119–128. 10.1016/j.appet.2012.12.018 23291218

[B44] Freeman LR, Granholm A-CE (2012) Vascular changes in rat hippocampus following a high saturated fat and cholesterol diet. J Cereb Blood Flow Metab 32:643–653. 10.1038/jcbfm.2011.168 22108721 PMC3318144

[B45] Friedman MJ, Keane TM, Resick PA (2014) Handbook of PTSD, Ed 2. New York: Guilford Publications.

[B46] Geyer MA, Swerdlow NR (2001) Measurement of startle response, prepulse inhibition, and habituation. Curr Protoc Neurosci Chapter 8:Unit8.7–Unit8.7. 10.1002/0471142301.ns0807s03 18428548

[B47] Gianlorenço ACL, Serafim KR, Canto-de-Souza A, Mattioli R (2012) Emotional memory consolidation impairment induced by histamine is mediated by H1 but not H2 receptors. Brain Res Bull 89:197–202. 10.1016/j.brainresbull.2012.09.003 22986235

[B48] Golub Y, Kaltwasser SF, Mauch CP, Herrmann L, Schmidt U, Holsboer F, Czisch M, Wotjak CT (2011) Reduced hippocampus volume in the mouse model of posttraumatic stress disorder. J Psychiatr Res 45:650–659. 10.1016/j.jpsychires.2010.10.014 21106206

[B49] Gorka AX, Hanson JL, Radtke SR, Hariri AR (2014) Reduced hippocampal and medial prefrontal gray matter mediate the association between reported childhood maltreatment and trait anxiety in adulthood and predict sensitivity to future life stress. Biol Mood Anxiety Disord 4:12 10.1186/2045-5380-4-12 25408863 PMC4236295

[B50] Goswami S, Cascardi M, Rodríguez-Sierra OE, Duvarci S, Paré D (2010) Impact of predatory threat on fear extinction in Lewis rats. Learn Mem 17:494–501. 10.1101/lm.1948910 20929713 PMC2948889

[B51] Goswami S, Samuel S, Sierra OR, Cascardi M, Paré D (2012) A rat model of post-traumatic stress disorder reproduces the hippocampal deficits seen in the human syndrome. Front Behav Neurosci 6:26 10.3389/fnbeh.2012.00026 22701407 PMC3372979

[B52] Grillon C, Baas J (2003) A review of the modulation of the startle reflex by affective states and its application in psychiatry. Clin Neurophysiol 114:1557–1579. 12948786 10.1016/s1388-2457(03)00202-5

[B53] Guo M, Huang T-Y, Garza JC, Chua SC, Lu X-Y (2013) Selective deletion of leptin receptors in adult hippocampus induces depression-related behaviours. Int J Neuropsychopharmacol 16:857–867. 10.1017/S1461145712000703 22932068 PMC3612133

[B54] Hall CS (1934) Emotional behavior in the rat. I. Defecation and urination as measures of individual differences in emotionality. J Comp Psychol 18:385–403. 10.1037/h0071444

[B55] Hall CS (1936) Emotional behavior in the rat. III. The relationship between emotionality and ambulatory activity. J Comp Psychol 22:345–352. 10.1037/h0059253

[B56] Hoeijmakers L, Harbich D, Schmid B, Lucassen PJ, Wagner KV, Schmidt MV, Hartmann J (2014) Depletion of FKBP51 in female mice shapes HPA axis activity. PLoS One 9:e95796. 10.1371/journal.pone.0095796 24759731 PMC3997427

[B57] Holz NE, Buchmann AF, Boecker R, Blomeyer D, Baumeister S, Wolf I, Rietschel M, Witt SH, Plichta MM, Meyer-Lindenberg A, Banaschewski T, Brandeis D, Laucht M (2015) Role of FKBP5 in emotion processing: results on amygdala activity, connectivity and volume. Brain Struct Funct 220:1355–1368. 10.1007/s00429-014-0729-5 24756342

[B58] Jacka FN, Cherbuin N, Anstey KJ, Sachdev P, Butterworth P (2015) Western diet is associated with a smaller hippocampus: a longitudinal investigation. BMC Med 13:215. 26349802 10.1186/s12916-015-0461-x PMC4563885

[B59] Jacobson L, Sapolsky R (1991) The role of the hippocampus in feedback regulation of the hypothalamic-pituitary-adrenocortical axis. Endocr Rev 12:118–134. 10.1210/edrv-12-2-118 2070776

[B60] Johannessen KB, Berntsen D (2013) Losing the symptoms: weight loss and decrease in posttraumatic stress disorder symptoms. J Clin Psychol 69:655–660. 10.1002/jclp.21962 23382106

[B61] Kalisch R, Schubert M, Jacob W, Kessler MS, Hemauer R, Wigger A, Landgraf R, Auer DP (2006) Anxiety and hippocampus volume in the rat. Neuropsychopharmacology 31:925–932. 10.1038/sj.npp.1300910 16192979

[B62] Karl A, Schaefer M, Malta LS, Dörfel D, Rohleder N, Werner A (2006) A meta-analysis of structural brain abnormalities in PTSD. Neurosci Biobehav Rev 30:1004–1031. 10.1016/j.neubiorev.2006.03.004 16730374

[B63] Kheirbek MA, Drew LJ, Burghardt NS, Costantini DO, Tannenholz L, Ahmari SE, Zeng H, Fenton AA, Hen R (2013) Differential control of learning and anxiety along the dorsoventral axis of the dentate gyrus. Neuron 77:955–968. 10.1016/j.neuron.2012.12.038 23473324 PMC3595120

[B64] Kjelstrup KG, Tuvnes FA, Steffenach H-A, Murison R, Moser EI, Moser M-B (2002) Reduced fear expression after lesions of the ventral hippocampus. Proc Natl Acad Sci USA 99:10825–10830. 10.1073/pnas.152112399 12149439 PMC125057

[B65] Klengel T, Mehta D, Anacker C, Rex-Haffner M, Pruessner JC, Pariante CM, Pace TWW, Mercer KB, Mayberg HS, Bradley B, Nemeroff CB, Holsboer F, Heim CM, Ressler KJ, Rein T, Binder EB (2013) Allele-specific FKBP5 DNA demethylation mediates gene-childhood trauma interactions. Nat Neurosci 16:33–41. 10.1038/nn.3275 23201972 PMC4136922

[B66] Krishna S, Keralapurath MM, Lin Z, Wagner JJ, La Serre de CB, Harn DA, Filipov NM (2015) Neurochemical and electrophysiological deficits in the ventral hippocampus and selective behavioral alterations caused by high-fat diet in female C57BL/6 mice. Neuroscience 297:170–181. 10.1016/j.neuroscience.2015.03.068 25849614

[B67] Kubzansky LD, Bordelois P, Jun HJ, Roberts AL, Cerda M, Bluestone N, Koenen KC (2014) The weight of traumatic stress: a prospective study of posttraumatic stress disorder symptoms and weight status in women. JAMA Psychiatry 71:44–51. 10.1001/jamapsychiatry.2013.2798 24258147 PMC4091890

[B68] Kühn S, Gallinat J (2013) Gray matter correlates of posttraumatic stress disorder: a quantitative meta-analysis. Biol Psychiatry 73:70–74. 10.1016/j.biopsych.2012.06.029 22840760

[B69] Labouesse MA, Stadlbauer U, Langhans W, Meyer U (2013) Chronic high fat diet consumption impairs sensorimotor gating in mice. Psychoneuroendocrinology 38:2562–2574. 10.1016/j.psyneuen.2013.06.003 23850224

[B70] Lanius RA, Vermetten E, Pain C (2010) The impact of early life trauma on health and disease. Cambridge, UK: Cambridge UP.

[B71] Lavebratt C, Aberg E, Sjöholm LK, Forsell Y (2010) Variations in FKBP5 and BDNF genes are suggestively associated with depression in a Swedish population-based cohort. J Affect Disord 125:249–255. 10.1016/j.jad.2010.02.113 20226536

[B72] Liao S-C, Lee M-B, Lee Y-J, Huang T-S (2004) Hyperleptinemia in subjects with persistent partial posttraumatic stress disorder after a major earthquake. Psychosom Med 66:23–28. 10.1097/01.PSY.0000106880.22867.0E 14747634

[B73] Maguen S, Madden E, Cohen B, Bertenthal D, Neylan T, Talbot L, Grunfeld C, Seal K (2013) The relationship between body mass index and mental health among Iraq and Afghanistan veterans. J Gen Intern Med 28 [Suppl 2]:S563–S570. 10.1007/s11606-013-2374-8 23807066 PMC3695271

[B74] Maniam J, Antoniadis CP, Le V, Morris MJ (2016) A diet high in fat and sugar reverses anxiety-like behaviour induced by limited nesting in male rats: impacts on hippocampal markers. Psychoneuroendocrinology 68:202–209. 10.1016/j.psyneuen.2016.03.007 26999723

[B75] McNay E (2015) Recurrent hypoglycemia increases anxiety and amygdala norepinephrine release during subsequent hypoglycemia. Front Endocrinol (Lausanne) 6:175. 10.3389/fendo.2015.00175 26635724 PMC4653740

[B76] McNeilly AD, Stewart CA, Sutherland C, Balfour DJK (2015) High fat feeding is associated with stimulation of the hypothalamic-pituitary-adrenal axis and reduced anxiety in the rat. Psychoneuroendocrinology 52:272–280. 10.1016/j.psyneuen.2014.12.002 25544739

[B77] Medina AM, Mejia VY, Schell AM, Dawson ME, Margolin G (2001) Startle reactivity and PTSD symptoms in a community sample of women. Psychiatry Res 101:157–169. 11286819 10.1016/s0165-1781(01)00221-9

[B78] Merikangas KR, He J-P, Burstein M, Swanson SA, Avenevoli S, Cui L, Benjet C, Georgiades K, Swendsen J (2010) Lifetime prevalence of mental disorders in U.S. adolescents: results from the national comorbidity survey replication–adolescent supplement (NCS-A). J Am Acad Child Adolesc Psychiatry 49:980–989. 20855043 10.1016/j.jaac.2010.05.017 PMC2946114

[B79] Meyza KZ, Boguszewski PM, Nikolaev E, Zagrodzka J (2011) Age increases anxiety and reactivity of the fear/anxiety circuit in Lewis rats. Behav Brain Res 225:192–200. 10.1016/j.bbr.2011.07.011 21782853

[B80] Mitchell KS, Aiello AE, Galea S, Uddin M, Wildman D, Koenen KC (2013) PTSD and obesity in the Detroit neighborhood health study. Gen Hosp Psychiatry 35:671–673. 10.1016/j.genhosppsych.2013.07.015 24035634 PMC3823753

[B81] Mondelli V, Cattaneo A, Belvederi Murri M, Di Forti M, Handley R, Hepgul N, Miorelli A, Navari S, Papadopoulos AS, Aitchison KJ, Morgan C, Murray RM, Dazzan P, Pariante CM (2011) Stress and inflammation reduce brain-derived neurotrophic factor expression in first-episode psychosis: a pathway to smaller hippocampal volume. J Clin Psychiatry 72:1677–1684. 10.4088/JCP.10m06745 21672499 PMC4082665

[B82] Moreno LA, Rodriguez G, Fleta J, Bueno-Lozano M, Lazaro A, Bueno G (2010) Trends of dietary habits in adolescents. Crit Rev Food Sci Nutr 50:106–112. 10.1080/10408390903467480 20112152

[B83] Morgan CA, Grillon C, Southwick SM, Davis M, Charney DS (1996) Exaggerated acoustic startle reflex in Gulf War veterans with posttraumatic stress disorder. Am J Psychiatry 153:64–68. 8540594 10.1176/ajp.153.1.64

[B84] Nilsson A, Radeborg K, Björck I (2009) Effects of differences in postprandial glycaemia on cognitive functions in healthy middle-aged subjects. Eur J Clin Nutr 63:113–120. 10.1038/sj.ejcn.1602900 17851459

[B85] Nugent NR, Ostrowski S, Christopher NC, Delahanty DL (2007) Parental posttraumatic stress symptoms as a moderator of child's acute biological response and subsequent posttraumatic stress symptoms in pediatric injury patients. J Pediatr Psychol 32:309–318. 10.1093/jpepsy/jsl005 16762993

[B86] Ogden CL, Lamb MM, Carroll MD, Flegal KM (2010) Obesity and socioeconomic status in children and adolescents: United States, 2005-2008. NCHS Data Brief 1–8. 21211166

[B87] Ornitz EM, Pynoos RS (1989) Startle modulation in children with posttraumatic stress disorder. Am J Psychiatry 146:866–870. 10.1176/ajp.146.7.866 2742011

[B88] Pagoto SL, Schneider KL, Bodenlos JS, Appelhans BM, Whited MC, Ma Y, Lemon SC (2012) Association of post-traumatic stress disorder and obesity in a nationally representative sample. Obesity (Silver Spring) 20:200–205. 10.1038/oby.2011.318 22016096

[B89] Pase CS, Roversi K, Trevizol F, Kuhn FT, Dias VT, Roversi K, Vey LT, Antoniazzi CT, Barcelos RCS, Bürger ME (2015) Chronic consumption of trans fat can facilitate the development of hyperactive behavior in rats. Physiol Behav 139:344–350. 10.1016/j.physbeh.2014.11.059 25433314

[B90] Pawlak CR, Ho Y-J, Schwarting RKW (2008) Animal models of human psychopathology based on individual differences in novelty-seeking and anxiety. Neurosci Biobehav Rev 32:1544–1568. 10.1016/j.neubiorev.2008.06.007 18619487

[B91] Pellow S, Chopin P, File SE, Briley M (1985) Validation of open:closed arm entries in an elevated plus-maze as a measure of anxiety in the rat. J Neurosci Methods 14:149–167. 2864480 10.1016/0165-0270(85)90031-7

[B92] Perkonigg A, Owashi T, Stein MB, Kirschbaum C, Wittchen H-U (2009) Posttraumatic stress disorder and obesity: evidence for a risk association. Am J Prev Med 36:1–8. 10.1016/j.amepre.2008.09.026 18976880

[B93] Pine DS, Cohen JA (2002) Trauma in children and adolescents: risk and treatment of psychiatric sequelae. Biol Psychiatry 51:519–531. 11950454 10.1016/s0006-3223(01)01352-x

[B94] Porsolt RD, Anton G, Blavet N, Jalfre M (1978) Behavioural despair in rats: a new model sensitive to antidepressant treatments. Eur J Pharmacol 47:379–391. 204499 10.1016/0014-2999(78)90118-8

[B95] Rasmussen DD, Crites NJ, Burke BL (2008) Acoustic startle amplitude predicts vulnerability to develop post-traumatic stress hyper-responsivity and associated plasma corticosterone changes in rats. Psychoneuroendocrinology 33:282–291. 10.1016/j.psyneuen.2007.11.010 18164825 PMC2291512

[B96] Ritov G, Richter-Levin G (2014) Water associated zero maze: a novel rat test for long term traumatic re-experiencing. Front Behav Neurosci 8:1. 10.3389/fnbeh.2014.00001 24478648 PMC3894455

[B97] Sapolsky RM (1986) Glucocorticoid toxicity in the hippocampus. Neuroendocrinology 43:440–444. 3736786 10.1159/000124561

[B98] Sapolsky RM (2001) Atrophy of the hippocampus in posttraumatic stress disorder: how and when? Hippocampus 11:90–91. 10.1002/hipo.1026 11345129

[B99] Sapolsky RM, Krey LC, McEwen BS (1984) Glucocorticoid-sensitive hippocampal neurons are involved in terminating the adrenocortical stress response. Proc Natl Acad Sci USA 81:6174–6177. 6592609 10.1073/pnas.81.19.6174 PMC391882

[B100] Schmidt U, Buell DR, Ionescu IA, Gassen NC, Holsboer F, Cox MB, Novak B, Huber C, Hartmann J, Schmidt MV, Touma C, Rein T, Herrmann L (2015) A role for synapsin in FKBP51 modulation of stress responsiveness: convergent evidence from animal and human studies. Psychoneuroendocrinology 52:43–58. 10.1016/j.psyneuen.2014.11.005 25459892

[B101] Schmiedt CW, Gogal RM, Harvey SB (2011) Biometric evidence of diet-induced obesity in Lew/Crl rats. Comp Med 6:131–137. PMC3079814 21535923

[B102] Schneider P, Ho Y-J, Spanagel R, Pawlak CR (2011) A novel elevated plus-maze procedure to avoid the one-trial tolerance problem. Front Behav Neurosci 5:43. 10.3389/fnbeh.2011.00043 21845176 PMC3146044

[B103] Scott KM, McGee MA, Wells JE, Oakley Browne MA (2008) Obesity and mental disorders in the adult general population. J Psychosom Res 64:97–105. 10.1016/j.jpsychores.2007.09.006 18158005

[B104] Sellbom KS, Gunstad J (2012) Cognitive function and decline in obesity. J Alzheimers Dis 30 [Suppl 2]:S89–S95. 10.3233/JAD-2011-111073 22258511

[B105] Sharma AN, Elased KM, Garrett TL, Lucot JB (2010) Neurobehavioral deficits in db/db diabetic mice. Physiol Behav 101:381–388. 10.1016/j.physbeh.2010.07.002 20637218 PMC3098504

[B106] Sharma S, Zhuang Y, Gomez-Pinilla F (2012) High-fat diet transition reduces brain DHA levels associated with altered brain plasticity and behaviour. Sci Rep 2:431. 10.1038/srep00431 22666534 PMC3362800

[B107] Shipton OA, El-Gaby M, Apergis-Schoute J, Deisseroth K, Bannerman DM, Paulsen O, Kohl MM (2014) Left-right dissociation of hippocampal memory processes in mice. Proc Natl Acad Sci USA 111:15238–15243. 10.1073/pnas.1405648111 25246561 PMC4210314

[B108] Shumake J, Barrett D, Gonzalez-Lima F (2005) Behavioral characteristics of rats predisposed to learned helplessness: reduced reward sensitivity, increased novelty seeking, and persistent fear memories. Behav Brain Res 164:222–230. 10.1016/j.bbr.2005.06.016 16095730

[B109] Sivanathan S, Thavartnam K, Arif S, Elegino T, McGowan PO (2015) Chronic high fat feeding increases anxiety-like behaviour and reduces transcript abundance of glucocorticoid signalling genes in the hippocampus of female rats. Behav Brain Res 286:265–270. 10.1016/j.bbr.2015.02.036 25721737

[B110] Smith BN, Tyzik AL, Neylan TC, Cohen BE (2015) PTSD and obesity in younger and older veterans: results from the mind your heart study. Psychiatry Res 229:895–900. 10.1016/j.psychres.2015.07.044 26210650 PMC4568132

[B111] Srivastava SK, Bhardwaj A, Arora S, Tyagi N, Singh AP, Carter JE, Scammell JG, Fodstad Ø, Singh S (2015) Interleukin-8 is a key mediator of FKBP51-induced melanoma growth, angiogenesis and metastasis. Br J Cancer 112:1772–1781. 10.1038/bjc.2015.154 25942396 PMC4647250

[B112] Stamm TJ, Rampp C, Wiethoff K, Stingl J, Mössner R, O’Malley G, Ricken R, Seemüller F, Keck M, Fisher R, Gaebel W, Maier W, Möller H-J, Bauer M, Adli M (2016) The FKBP5 polymorphism rs1360780 influences the effect of an algorithm-based antidepressant treatment and is associated with remission in patients with major depression. J Psychopharmacol 30:40–47. 10.1177/0269881115620459 26645208

[B113] Stern CAJ, Do Monte FHM, Gazarini L, Carobrez AP, Bertoglio LJ (2010) Activity in prelimbic cortex is required for adjusting the anxiety response level during the elevated plus-maze retest. Neuroscience 170:214–222. 10.1016/j.neuroscience.2010.06.080 20620194

[B114] Strachan MW, Deary IJ, Ewing FM, Frier BM (2000) Recovery of cognitive function and mood after severe hypoglycemia in adults with insulin-treated diabetes. Diabetes Care 23:305–312. 10868856 10.2337/diacare.23.3.305

[B115] Treit D, Menard J, Royan C (1993) Anxiogenic stimuli in the elevated plus-maze. Pharmacol Biochem Behav 44:463–469. 8446680 10.1016/0091-3057(93)90492-c

[B116] Turner PV, Vaughn E, Sunohara-Neilson J, Ovari J, Leri F (2012) Oral gavage in rats: animal welfare evaluation. J Am Assoc Lab Anim Sci 51:25–30. 22330864 PMC3276962

[B117] Tyree SM, Munn RGK, McNaughton N (2016) Anxiolytic-like effects of leptin on fixed interval responding. Pharmacol Biochem Behav 148:15–20. 10.1016/j.pbb.2016.05.005 27180106

[B118] van Rooij SJH, Kennis M, Sjouwerman R, van den Heuvel MP, Kahn RS, Geuze E (2015) Smaller hippocampal volume as a vulnerability factor for the persistence of post-traumatic stress disorder. Psychol Med 45:2737–2746. 10.1017/S0033291715000707 25936409

[B119] Veenema AH, Meijer OC, de Kloet ER, Koolhaas JM (2003) Genetic selection for coping style predicts stressor susceptibility. Journal of Neuroendocrinology 15:256–267. 12588514 10.1046/j.1365-2826.2003.00986.x

[B120] Vollmayr B, Bachteler D, Vengeliene V, Gass P, Spanagel R, Henn F (2004) Rats with congenital learned helplessness respond less to sucrose but show no deficits in activity or learning. Behav Brain Res 150:217–221. 10.1016/S0166-4328(03)00259-6 15033295

[B121] Wakabayashi C, Numakawa T, Ooshima Y, Hattori K, Kunugi H (2015) Possible role of the dopamine D1 receptor in the sensorimotor gating deficits induced by high-fat diet. Psychopharmacology (Berl) 232:4393–4400. 10.1007/s00213-015-4068-x 26359228

[B122] Wang Y, Xu X-Y, Feng C-H, Li Y-L, Ge X, Zong G-L, Wang Y-B, Feng B, Zhang P (2015) Patients with type 2 diabetes exhibit cognitive impairment with changes of metabolite concentration in the left hippocampus. Metab Brain Dis 30:1027–1034. 10.1007/s11011-015-9670-4 25875132 PMC4491369

[B123] Woon FL, Hedges DW (2008) Hippocampal and amygdala volumes in children and adults with childhood maltreatment-related posttraumatic stress disorder: a meta-analysis. Hippocampus 18:729–736. 10.1002/hipo.20437 18446827

[B124] Woon FL, Sood S, Hedges DW (2010) Hippocampal volume deficits associated with exposure to psychological trauma and posttraumatic stress disorder in adults: a meta-analysis. Prog Neuropsychopharmacol Biol Psychiatry 34:1181–1188. 10.1016/j.pnpbp.2010.06.016 20600466

[B125] Yang L, Isoda F, Yen K, Kleopoulos SP, Janssen W, Fan X, Mastaitis J, Dunn-Meynell A, Levin B, McCrimmon R, Sherwin R, Musatov S, Mobbs CV (2012) Hypothalamic Fkbp51 is induced by fasting, and elevated hypothalamic expression promotes obese phenotypes. Am J Physiol Endocrinol Metab 302:E987–E991. 10.1152/ajpendo.00474.2011 22318949 PMC3330722

[B126] Yehuda R, Teicher MH, Seckl JR, Grossman RA, Morris A, Bierer LM (2007) Parental posttraumatic stress disorder as a vulnerability factor for low cortisol trait in offspring of holocaust survivors. Arch Gen Psychiatry 64:1040–1048. 10.1001/archpsyc.64.9.1040 17768269

[B127] Yehuda R, Daskalakis NP, Bierer LM, Bader HN, Klengel T, Holsboer F, Binder EB (2016) Holocaust exposure induced intergenerational effects on FKBP5 methylation. Biol Psychiatry 80:372–380. 26410355 10.1016/j.biopsych.2015.08.005

[B128] Young KA, Thompson PM, Cruz DA, Williamson DE, Selemon LD (2015) BA11 FKBP5 expression levels correlate with dendritic spine density in postmortem PTSD and controls. Neurobiol Stress 2:67–72. 10.1016/j.ynstr.2015.07.002 26844242 PMC4721476

[B129] Zobel A, Schuhmacher A, Jessen F, Höfels S, Widdern von O, Metten M, Pfeiffer U, Hanses C, Becker T, Rietschel M, Scheef L, Block W, Schild HH, Maier W, Schwab SG (2010) DNA sequence variants of the FKBP5 gene are associated with unipolar depression. Int J Neuropsychopharmacol 13:649–660. 10.1017/S1461145709991155 20047716

